# In Situ Engineering of Tumor Cells as Self‐Sustaining cDC1 Programming Factories for Effective Cancer Immunotherapy

**DOI:** 10.1002/advs.202508632

**Published:** 2025-11-03

**Authors:** Shuiling Jin, Xiaoxi Wang, Bingyu Li, Xueqin Zhu, Zimai Liu, Jiao Lu, Yuanyuan Wei, Zixian Wu, Kai Li, Tiantian Zhang, Zonghong He, Pingping Zhu, Yuanming Qi, Benyu Liu, Hui Liu, Yongchao Wang, Zhenzhen Chen

**Affiliations:** ^1^ Department of Oncology The First Affiliated Hospital of Zhengzhou University Zhengzhou 450052 China; ^2^ School of Life Sciences Zhengzhou University Zhengzhou 450001 China; ^3^ Henan Key Laboratory of Bioactive Macromolecules Zhengzhou University Zhengzhou 450001 China; ^4^ International Joint Laboratory for Protein and Peptide Drugs of Henan Province Zhengzhou University Zhengzhou 450001 China; ^5^ Academy of Medical Sciences Zhengzhou University Zhengzhou 450001 China; ^6^ School of Medicine Shihezi University Shihezi 832003 China

**Keywords:** drug delivery, extracellular vesicles, gene delivery, immunotherapy, nanoparticles

## Abstract

Conventional type 1 dendritic cells (cDC1s) play a crucial role in initiating and regulating antitumor immunity. However, the insufficient infiltration and dysfunctional state of these cells within the tumor microenvironment hinder antitumor immune response. A tumor‐derived extracellular vesicle (EV), termed AS16‐EL@MPLA/p‐FX, is designed to engineer tumor cells into cDC1 programming factories. The EV lumen carries a plasmid encoding XCL1 and FLT3L. Leveraging the prolonged circulation time and homotypic targeting properties of tumor‐derived EV, AS16‐EL@MPLA/p‐FX demonstrates enhanced tumor‐selective accumulation and efficient cellular internalization. Following uptake, tumor cells are reprogrammed into in situ cytokine factories that continuously secrete XCL1 and FLT3L, which effectively recruit and differentiate cDC1s within the tumor microenvironment. Simultaneously, MPLA embedded in the EV membrane is released locally and activates the newly accumulated cDC1s through the TLR4 pathway. Furthermore, an AS16 peptide is tethered to the EV surface via a matrix metalloproteinase‐2–cleavable linker. The enzymatic release of AS16 disrupts the VEGF–NRP1 interaction, preventing cDC1 exhaustion. The engineered EV, AS16‐EL@MPLA/p‐FX, exhibited remarkable tumor‐targeting capabilities, promoting the recruitment, differentiation, and activation of cDC1s. This innovative approach not only significantly inhibited tumor growth but also triggered a robust immune memory response, safeguarding against tumor metastasis and recurrence.

## Graphical abstract

The fused EV platform AS16‐EL@MPLA/p‐FX reprograms tumor cells into cytokine‐producing factories that continuously secrete XCL1 and FLT3L to recruit and differentiate cDC1s, while embedded MPLA activates them through TLR4 signaling and AS16 prevents their exhaustion, thereby enhancing antitumor immunity and establishing durable immune memory against tumor progression, metastasis, and recurrence.

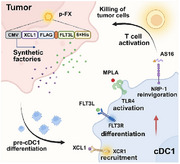

## 1 Introduction

The development of cancer immunotherapies, including checkpoint inhibitors, CAR‐T cell therapies, and cancer vaccines, has shown great promise for a variety of cancers.^[^ ^1^, ^2^, ^3^ ^]^ However, immunotherapies are still confronted with challenges of low response rate and immune resistance.^[^ ^4^, ^5^, ^6^ ^]^ To achieve effective immune responses, tumor antigens have to be effectively presented to T cells and elicit antigen‐specific immune responses. Dendritic cells (DCs) play a crucial role in antigen presentation and initiation of antitumor immune responses.^[^ ^7^, ^8^ ^]^ However, DCs are often excluded from the TME and fail to prime efficient antitumor immunity. Various DC‐based therapeutic strategies, including ex vivo‐generated DC vaccines and in vivo DC recruitment, have been well developed.^[^ ^9^, ^10^, ^11^, ^12^ ^]^ However, their therapeutic efficacy remains limited. A primary obstacle is the functional heterogeneity of DC subsets, which differ markedly in antigen‐processing and T‐cell‐priming capacity.^[^ ^13^, ^14^ ^]^ DCs are classified as monocyte‐derived DC (MoDC), plasmacytoid DCs (pDC), type 1 conventional DCs (cDC1), and type 2 conventional DCs (cDC2).^[^ ^15^ ^]^ Among all the DC subsets, cDC1 is the only subset capable of robust cross‐presentation of tumor antigens and subsequent activation of cytotoxic CD8⁺ T cells.^[^ ^16^, ^17^, ^18^ ^]^ Current DC‐based approaches fail to sufficiently enrich and activate cDC1s, leading to inadequate antigen presentation and attenuated antitumor immunity. Consequently, strategies that selectively enhance the infiltration and functional competence of cDC1s are crucial for improving the clinical outcomes of cancer immunotherapy.

Studies have demonstrated that Fms‐like tyrosine kinase 3 ligand (FLT3L) can drive progenitor cells to differentiate into cDC1s,^[^ ^19^, ^20^ ^]^ whereas X‐C motif chemokine ligand 1 (XCL1) could selectively attract cDC1s through the XCR1–XCL1 axis.^[^ ^21^, ^22^ ^]^  However, systemic administration of FLT3L or XCL1 is hampered by dose‐dependent toxicity and rapid plasma clearance. This makes it difficult to sustain therapeutically effective concentrations within the tumor microenvironment, thus achieving limited enrichment of cDC1s.  Moreover, recent studies indicated that cDC1s can be inactivated or exhausted by the interaction between vascular endothelial growth factor (VEGF) and neuropilin 1 (NRP‐1) expressed on cDC1s.^[^ ^23^, ^24^, ^25^ ^]^ Therefore, the antitumor activity of cDC1s relies not only on their adequate recruitment and differentiation but also on maintaining their activation state in the TME. This process requires the coordinated action of multiple cytokines to reprogram cDC1 for effective antitumor immunity.

In recent years, the development of synthetic biology has offered new opportunities for the precise modulation of cytokine production within the TME. Through programmable genetic circuits of synthetic biology,^[^ ^26^, ^27^ ^]^ cytokines can be precisely released with exquisite spatiotemporal control, enabling the fine‑tuned recruitment, differentiation, and activation of specific immune cells.^[^ ^28^, ^29^ ^]^ However, current strategies predominantly employ engineered bacteria to synthesize cytokines, which may cause toxicity arising from bacterial immunogenicity and off‐target inflammation.^[^ ^30^, ^31^ ^]^ An alternative approach is to engineer tumor cells into “synthetic factories” capable of controllable cytokine secretion without introducing exogenous microorganisms.^[^ ^32^, ^33^ ^]^ This strategy holds promise for continuously shaping a favorable immune environment for cDC1 cells while effectively reducing safety risks.

**Scheme 1:**
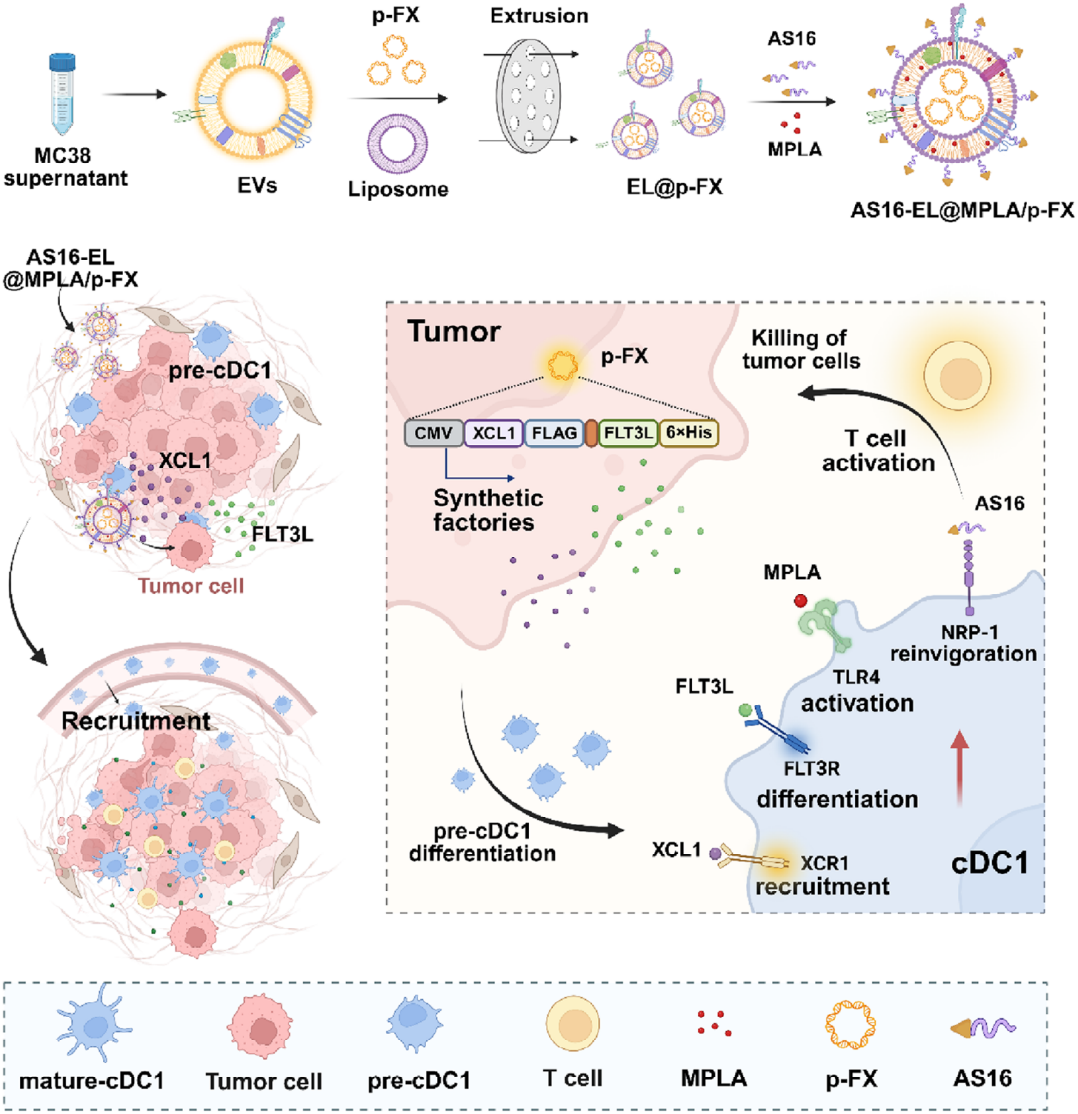
Schematic illustration of the construction and antitumor mechanism of AS16‐EL@MPLA/p‐FX. 1) Recruitment and differentiation of cDC1s. With the homologous targeting function of tumor‐cell‐membrane components, when AS16‐EL@MPLA/p‐FX enters the tumor microenvironment, the AS16 peptide on its surface is cleaved and released by the action of the MMP2 enzyme. XCL1 and FLT3L are expressed after the endocytosis of AS16‐EL@MPLA/p‐FX by tumor cells. A concentration gradient of the chemokine XCL1 forms at the tumor site to recruit cDC1s into the tumor microenvironment. Pre‐cDC1s in the tumor differentiate into cDC1s under the influence of FLT3L. 2) Disinhibition and activation of cDC1s. NRP1 on the cDC1s is competitively bound by the AS16 peptide to block the inhibition of cDC1s by VEGF. At the same time, the MPLA on AS16‐EL@MPLA/p‐FX reactivates the cDC1s. The activated cDC1s stimulate the immune response of CD8^+^ T cells. Tumor cells are killed by the IFN‐γ secreted by CD8^+^ T cells. Tumor antigens are released after tumor cell death. The released tumor antigens are ingested by cDC1s and presented to CD8^+^ T cells.

In this study, we designed a tumor‐derived extracellular vesicle (EV), termed AS16‐EL@MPLA/p‐FX, to engineer tumor cells into cDC1 programming factories (**Scheme** **1**). The EV lumen carries a plasmid encoding XCL1 and FLT3L. Owing to the prolonged circulation time and homotypic targeting of tumor‐derived EVs, AS16‐EL@MPLA/p‐FX accumulates efficiently within the tumor tissue and is readily internalized by tumor cells. Subsequently, these tumor cells are reprogrammed into in situ cytokine factories that continuously secrete XCL1 and FLT3L, which effectively recruit and differentiate cDC1s within the tumor microenvironment. Concurrently, monophosphoryl lipid A (MPLA) embedded in the EV membrane is liberated locally and activates the newly accumulated cDC1s through the TLR4 pathway. To prevent cDC1 exhaustion, an AS16 peptide is tethered to the EV surface via a matrix metalloproteinase‐2 (MMP‐2)‐cleavable linker.^[^ ^34^ ^]^ The enzymatic release of AS16 disrupts the VEGF–NRP1 interaction, preventing cDC1 exhaustion. This integrated platform sequentially enriches, activates, and preserves cDC1s, eliciting potent cytotoxic T‐cell responses that suppress primary tumor growth and establish durable immune memory to mitigate metastasis and recurrence.

## 2 Results and Discussion

### 2.1 Preparation and Characterization of AS16‐EL@MPLA/p‐FX

**Figure 1:**
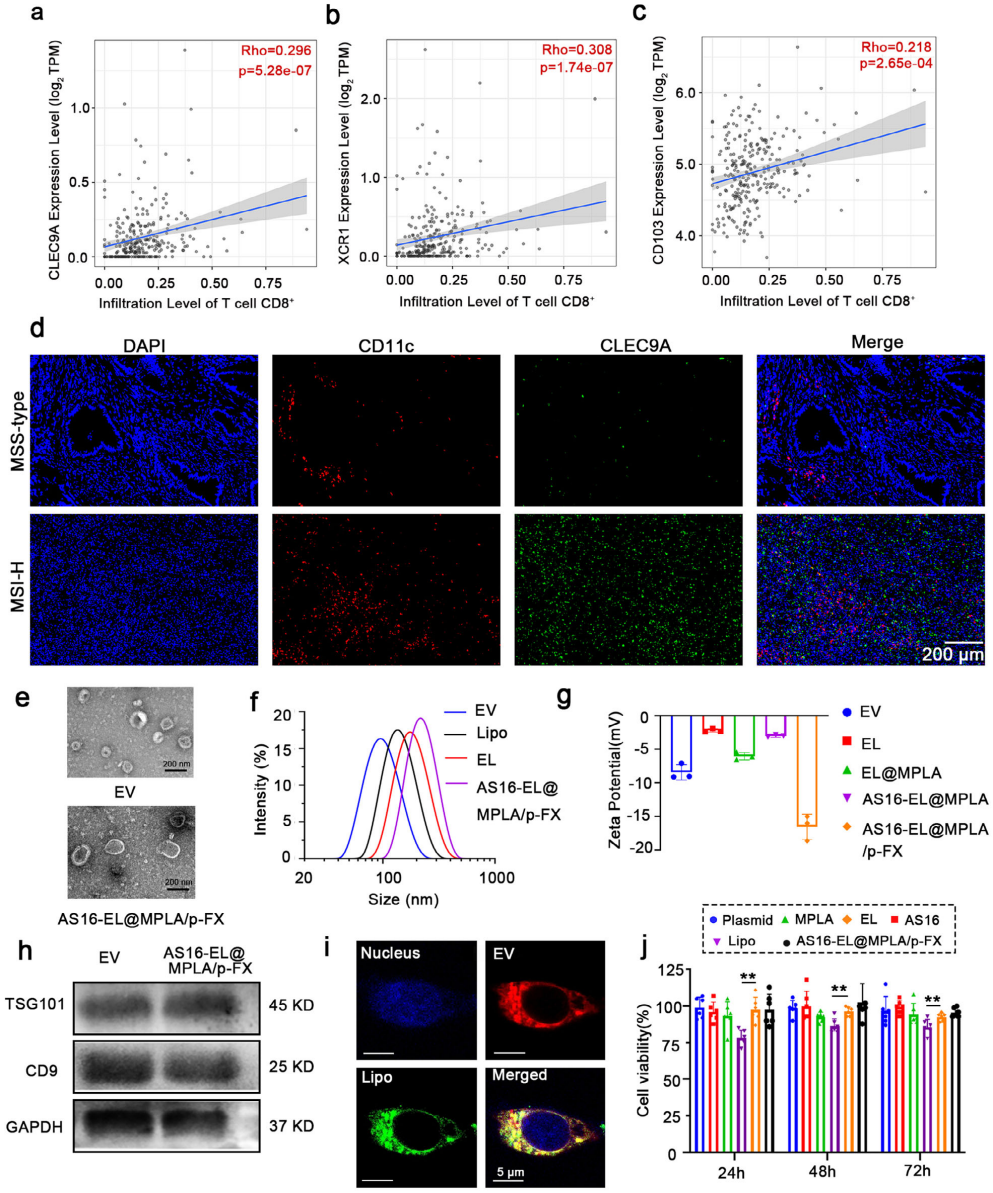
Characterization of AS16‐EL@MPLA/p‐FX. a–c) Correlation analysis between CLEC9A (a), XCR1 (b), and CD103 (c) expression and CD8^+^ T cell infiltration levels in pancreatic cancer. d) Immunofluorescence detection of cDC1 infiltration. scale bar: 200 µm. e) TEM images of the EVs and the AS16‐EL@MPLA/p‐FX fused EVs. scale bar: 200 nm. f) Particle size and g) potential changes in AS16‐EL@MPLA/p‐FX during its preparation, respectively. Data are expressed as means ± SD, *n* = 3. h) Integrity of the membrane protein on AS16‐EL@MPLA/p‐FX was characterized using western blotting. i) Fusion effect of AS16‐EL@MPLA/p‐FX was detected using confocal microscopy. Dye labeling: nuclei: Hoechst 33342; EV: DiO; Lipo: DiD; scale bar: 5 µm. j) Viability of MC38 cells treated with different drugs, *n* = 6. Cell viability (%) = (optical density [OD]_experimental group_ /OD_control group_) × 100%. Data are means ± SD, *n* = 3; ^**^ *p* < 0.01.

CLEC9A, XCR1, and CD103 are characteristic marker genes of cDC1. cDC1 plays a key role in anti‐tumor immunity, activating CD8^+^ T cells mainly through cross‐presentation of antigens and promoting their infiltration into tumor tissue. Our bioinformatics analysis showed that in colon cancer, the gene expression levels of CLEC9A, XCR1, and CD103 were all significantly and positively correlated with the degree of CD8^+^ T cell infiltration in tumor tissues (**Figure** **1**a–c). This finding suggests that the enrichment of cDC1 in the tumor microenvironment may promote the recruitment and activation of CD8^+^ T cells, thereby enhancing the anti‐tumor immune response in “cold tumors.” Notably, in typical immune “cold tumors” such as pancreatic and breast cancers, we also observed a significant positive correlation between the expression of the cDC1 signature gene and CD8^+^ T cell infiltration (Figure S1a–c,g–i, Supporting Information). Further survival analysis showed that in patients, high expression of these genes was significantly associated with better survival prognosis (Figure S1d–f,j,k, Supporting Information). By quantitative analysis of immunofluorescence staining, we observed a significant increase in the density of cDC1 infiltration within the tumor tissues of MSI‐H patients compared to MSS‐type patients, suggesting that MSI‐H tumors may have a stronger immune cell recruiting ability or a more active antigen‐presenting microenvironment (Figure 1d). In this study, we constructed an engineered EV for the tumor‐targeted co‐delivery of a plasmid and immunomodulators to modulate the function of cDC1s. First, a plasmid encoding XCL1 and FLT3L was designed and synthesized. The plasmid and MPLA were then loaded into the tumor‐cell‐derived EV (isolated from the culture supernatant) using a lipid fusion method to form the fused EV (EL@MPLA/p‐FX).^[^ ^35^, ^36^, ^37^ ^]^ Utilizing the Fmoc solid‐phase peptide synthesis method, our experiment successfully synthesized a fatty acid chain‐modified MMP‐2‐responsive peptide, C16‐RRKKPLGLAG‐AS16 (Figure S2a,b, Supporting Information). In this construct, the C16 moiety inserts into the hydrophobic cavity of the phospholipid bilayer of the fused EV, serving to anchor the AS16 peptide onto the fused EV. The RRKK sequence, consisting of positively charged amino acids, engages in electrostatic attraction with the negatively charged EV membrane, further facilitating the immobilization of AS16 on the fused EV. To verify whether the synthesized C16‐RRKKPLGLAG‐AS16 peptide can be cleaved by MMP‐2, we conducted an enzymatic cleavage experiment and identified the cleavage products through mass spectrometry. In the presence of MMP‐2, the C16‐RRKKPLGLAG‐AS16 peptide was successfully cleaved (Figure S2c, Supporting Information), resulting in the formation of a C16‐RRKKPLG fragment with a molecular weight of 1092 Da and a LAG‐AS16 fragment with a molecular weight of 1924 Da. In addition, the HPLC results showed that two peaks of product peptides appeared in the C16‐RRKK‐PLGLAG‐AS16 + MMP2 group, and their retention times were consistent with those of the LAG‐AS16 and C16‐RRKK‐PLG standards. Notably, a time‐dependent increase in peak area was observed, with a significant rise at 3 h, further confirming the MMP2‐mediated release of these two functional peptides (Figure S2d, Supporting Information).

EVs and liposomes can form hybrid EVs through fusion. Transmission electron microscopy (TEM) results showed that EVs exhibited a characteristic saucer‐like shape with a diameter of ≈90 nm, while the AS16‐EL@MPLA/p‐FX exhibit a slightly larger particle size (**≈**160 nm) with a lipid vesicle‐like morphology (Figure 1e). Dynamic light scattering (DLS) revealed that the diameter of the EL (≈160 nm) was slightly larger than that of Lipo2000 (≈120 nm) and EVs (≈90 nm), which may be due to the membrane fusion process. Furthermore, AS16‐EL@MPLA/p‐FX exhibits a larger hydrodynamic diameter of ≈220 nm compared to the EL. It may be attributed to the enhanced surface negative charge caused by MPLA/p‐FX, which increases electrostatic repulsion and thus the apparent hydrodynamic size (Figure 1f). The zeta potentials were evaluated during the preparation of the EV, and that of AS16‐EL@MPLA/p‐FX was lower than that of EL, which was attributed to the loading of the negatively charged plasmid (Figure 1g). The membrane proteins, such as CD9 and TSG101 carried by EVs can well promote the transportation of EVs. Through Western blot verification, after the preparation of fused EVs, the integrity of their membrane proteins is maintained, indicating that membrane proteins were not lost during the preparation process (Figure 1h). To further confirm the successful preparation of the AS16‐EL@MPLA/p‐FX, we labeled EV with DiD (red) and Lipo with DiO (green). Significant colocalization of DiD and DiO fluorescence was observed using confocal laser scanning microscopy (CLSM), indicating that EL and Lipo fused well to form intact nanoparticles (Figure 1i). The efficiency of encapsulation of the plasmid was quantified using UV–vis absorption spectroscopy. The AS16 peptide was quantified using liquid chromatography (LC)‐MS, and MPLA was quantified using a LAL assay, according to the manufacturer's protocol. The encapsulation efficiencies of the plasmid, AS16 peptide, and MPLA were confirmed to be 63.93% ± 2.92%, 43.05% ± 4.05% and 37.93% ± 3.05%, respectively, and the loading ratios were 31.97% ± 1.79%, 4.30% ± 0.50%, and 0.38% ± 0.03%, respectively (Table S1, Supporting Information). Cell viability was evaluated using an MTT (3‐(4,5‐Dimethylthiazol‐2‐yl)‐2,5‐diphenyltetrazolium bromide) assay to verify the toxicity of the nanoparticle. As shown in Figure 1j, all the formulations were nontoxic to tumor cells. Hemolysis assays were conducted to further evaluate the blood compatibility of the nanoparticle; the hybrid EVs exhibited significantly lower hemolytic toxicity compared to the AS16‐Lipo@MPLA/p‐FX, which used the lipo2000 in place of the hybrid EVs as a nanoparticle (Figure S3, Supporting Information). Notably, the hybrid EVs had attenuated toxic effects compared with those of Lipo.

### 2.2 Recruitment of cDC1s by AS16‐EL@MPLA/p‐FX

**Figure 2:**
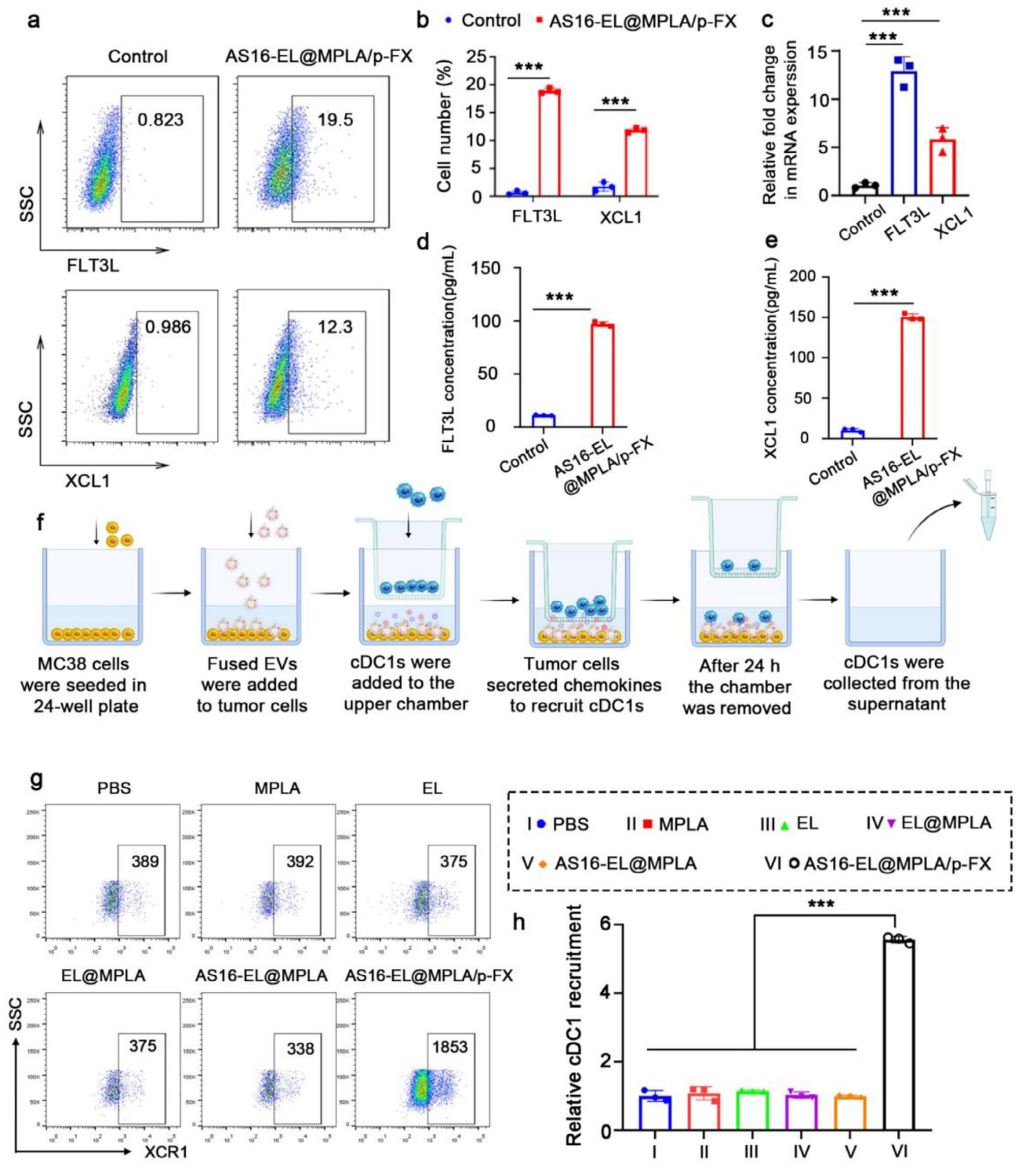
Recruitment of cDC1s by AS16‐EL@MPLA/p‐FX. a,b) Representative flow cytometric analysis and quantification of the expression of XCL1 and FLT3L in MC38 cells transfected with AS16‐EL@MPLA/p‐FX. c) The mRNA expression levels of XCL1 and FLT3L in MC38 cells. d, e) The secretion of FLT3L and XCL1 from MC38 cells. f) Schematic diagram of the recruitment assay. Using the transwell system, MC38 cells were treated with AS16‐EL@MPLA/p‐FX in the lower chamber, and the DCs in the upper chamber crossed the filter membrane into the lower chamber in the presence of secreted chemokines. We collected the cells in the lower chamber and counted them using flow cytometry to characterize their ability to recruit cDC1s. g) Representative flow cytometry plots showing the number of XCR1⁺ cells in the lower chamber, indicating the recruitment of cDC1s under different treatments. h) Quantitative analysis of cDC1 recruitment, expressed as fold change relative to the PBS group. Data are means ± SD, *n* = 3; ^*^ *p* < 0.05, ^**^ *p* < 0.01, ^***^ *p* < 0.001, ns: no significant difference.

The above results have proven the successful construction of AS16‐EL@MPLA/p‐FX. Next, we will further validate whether AS16‐EL@MPLA/p‐FX can successfully transfect MC38 cells and express XCL1 and FLT3L. These two cytokines, XCL1 and FLT3L, aid in the recruitment and expansion of cDC1 at tumor sites. Compared to the empty plasmid group, the group with the expression plasmid successfully expressed both XCL1 and FLT3L in MC38 cells (**Figure** **2**a,b). The mRNA levels of FLT3L and XCL1 were evaluated, and the results demonstrated that cells treated with AS16‐EL@MPLA/p‐FX exhibited significantly increased expression (Figure 2c). ELISA analysis confirmed the cytokine‐promoting effect of AS16‐EL@MPLA/p‐FX, revealing that XCL1 and FLT3L secretion levels in the supernatant were increased 14.2 and 8.5‐fold, respectively, relative to the control group (Figure 2d,e). In the subsequent experiments, cDC1 recruitment by AS16‐EL@MPLA/p‐FX was investigated using a transwell system (Figure 2f). In the transwell assay, we added cDC1 cells that had been induced for 12 days, while the lower chamber contained MC38 cells transfected with AS16‐EL@MPLA/p‐FX. After 24 h, we collected the medium from the lower chamber and used flow cytometry to detect the proportion of cDC1 cells that migrated to the lower chamber in different groups. The results indicated that only AS16‐EL@MPLA/p‐FX treatment effectively induced cDC1 migration, recruiting approximately five times more cells than the PBS control. No significant differences were observed between the other experimental groups (lacking p‐FX) and the PBS group (Figure 2g,h). These data conclusively demonstrate that AS16‐EL@MPLA/p‐FX efficiently drives the expression and secretion of therapeutic cytokines and specifically enhances the recruitment of cDC1 to tumor sites.

### 2.3 AS16‐EL@MPLA/p‐FX Blocks the Interaction Between VEGF and NRP1, Promoting cDC1s Maturation

**Figure 3:**
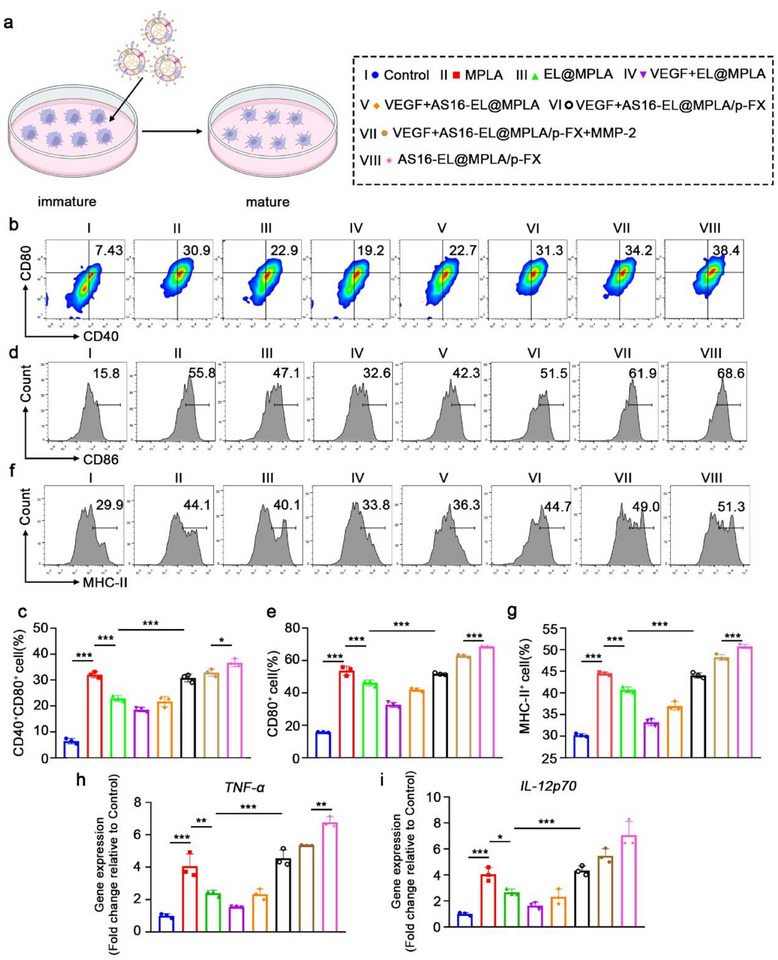
Activation of cDC1s by AS16‐EL@MPLA/p‐FX. a) Schematic of DC maturation. b–g) After cDC1s were treated with different formulations for 24 h, they were collected and analyzed using flow cytometry to detect CD40, CD80, CD86, and MHC‐II on their surfaces. h) TNF‐α and (i) IL‐12p70 expression by cDC1 were quantified using RT‐qPCR. Data are means ± SD, *n* = 3; ^*^ *p* < 0.05, ^**^ *p* < 0.01, ^***^ *p* < 0.001, ns: no significant difference.

It has been reported that cDC1s are functionally impaired or exhausted through the interaction between tumor‐derived VEGF and NRP1 expressed on cDC1s.^[^ ^38^, ^39^ ^]^ To investigate whether AS16‐EL@MPLA/p‐FX can block VEGF–NRP1 binding and promote cDC1 activation, we treated FLT3L‐induced cDC1 cells (12‐day culture) with different formulations. Flow cytometry analysis showed that, compared with the control group (7.43%), treatment with MPLA alone led to cDC1 activation (30.9%). Notably, the AS16‐EL@MPLA/p‐FX group demonstrated a significantly higher activation level (38.4%), underscoring the critical role of AS16 in effectively blocking the VEGF–NRP1 interaction and thereby further enhancing cDC1 activation. However, this activation induced by AS16‐EL@MPLA/p‐FX alone was suppressed in the presence of VEGF (31.3%). Notably, upon MMP‐2–mediated cleavage that triggered AS16 peptide release, AS16‐EL@MPLA/p‐FX effectively counteracted VEGF‐induced suppression (**Figure** **3**b,c). Consistent trends were observed in the expression of CD86 and MHC‐II on cDC1s (Figure 3d–g), indicating that AS16‐EL@MPLA/p‐FX effectively disrupts VEGF‐NRP1 interaction and promotes cDC1 maturation. RT‐qPCR analysis further revealed significant upregulation of TNF‐α (Figure 3h) and IL‐12p70 (Figure 3i) in the AS16‐EL@MPLA/p‐FX group following MMP‐2 treatment. These findings underscore the potential of AS16‐EL@MPLA/p‐FX, particularly upon MMP2‐triggered activation, as a promising strategy for antitumor immunotherapy.

### 2.4 AS16‐EL@MPLA/p‐FX Facilitated the Proliferation of and Production of Interferon γ (IFN‐γ) by CD8^+^ T Cells

CD8^+^ T cell activation and proliferation are crucial for an efficient immune response.^[^ ^40^ ^]^ Therefore, we investigated whether the maturation of cDC1s induced by AS16‐EL@MPLA/p‐FX facilitated the proliferation of CD8^+^ T cells. cDC1s treated with different formulations were cocultured with CD8^+^ T cells labeled with carboxyfluorescein succinimidyl ester (CFSE). Flow cytometry was used to evaluate the proliferation of the CD8^+^ T cells. The proliferation in the MPLA group (5.09%) was not significantly greater than that in the control group (4.31%). This may be due to the absence of the first stimulatory signal resulting from the lack of antigen stimulation. After the addition of EL@MPLA, antigen stimulation was provided, leading to a significant increase in CD8^+^ T cell proliferation (13.1%). Meanwhile, in the presence of VEGF, the addition of EL@MPLA did not enhance CD8^+^ T cell proliferation (5.66%), possibly due to the suppression of the second stimulatory signal generated by cDC1 activation, preventing cDC1 from properly stimulating the proliferation and differentiation of CD8^+^ T cells. However, in the AS16‐EL@MPLA group, CD8^+^ T cell proliferation was restored (16.9%), indicating that the AS16 peptide relieved the inhibitory effect of VEGF on cDC1. More importantly, in the AS16‐EL@MPLA/p‐FX group, CD8^+^ T cell proliferation further increased (23.3%), which may be attributed to the supportive role of the growth factor FLT3L on cDC1. Finally, in the AS16‐EL@MPLA/p‐FX group with the addition of MMP‐2, CD8^+^ T cell proliferation was the most significant (38.9%) (Figure S4, Supporting Information). An ELISA was used to examine the secretion of IFN‐γ by the CD8^+^ T cells, and the results were consistent with the trends in the proliferation of CD8^+^ T cells (Figure S5, Supporting Information). These results demonstrate that AS16‐EL@MPLA/p‐FX stimulates CD8^+^ T‐cell proliferation and IFN‐γ production.

### 2.5 Homologous Targeting and Biodistribution of AS16‐EL@MPLA/p‐FX In Vivo

**Figure 4:**
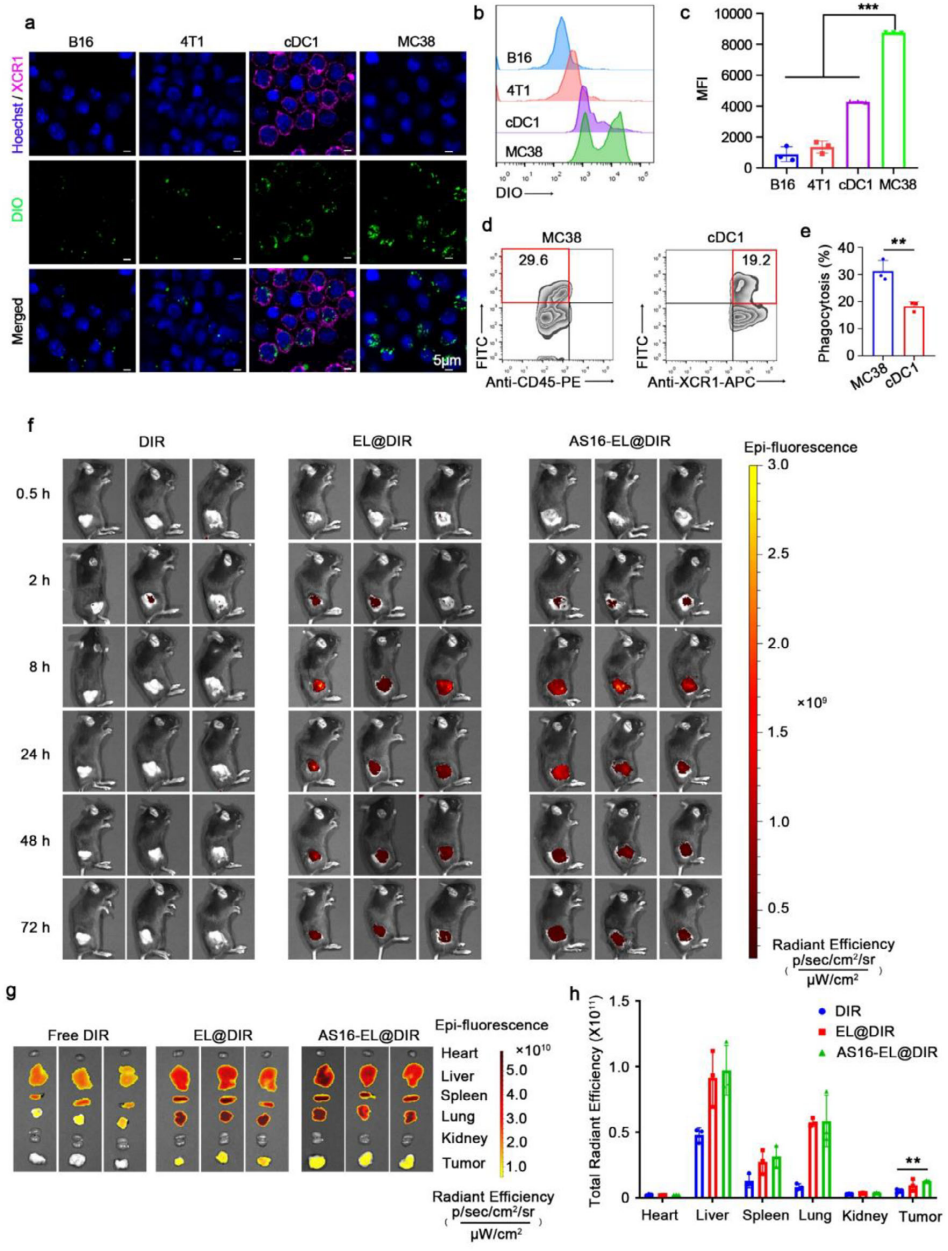
Tumor targeting ability of AS16‐EL@MPLA/p‐FX in vitro and in vivo. a) MC38, cDC1, B16, and 4T1 cells were incubated with DIO‐labeled AS16‐EL@MPLA/p‐FX (100 µg mL^−1^) for 3 h and then stained with Hoechst 33342. The phagocytosis of the AS16‐EL@MPLA/p‐FX by the different cell lines was observed using CLSM. Scale bar: 5 µm. b,c) Flow‐cytometric analysis of the different cell lines incubated with DIO‐labeled nanoparticles. d,e) In vivo uptake efficiency of AS16‐EL@MPLA/p‐FX nanoparticles by cDC1 and MC38 cells. DIO‐labeled AS16‐EL@MPLA/p‐FX nanoparticles were detected in the FITC channel, and their cellular uptake within tumor tissues was assessed by flow cytometry. f)Fluorescence images of the MC38 tumor‐bearing mice at 0.5, 2, 8, 24, 48, 72 h post‐injection of free DIR, EL@DIR, and AS16‐EL@DIR. g,h) Mean fluorescence intensity of organs and tumors 72 h after intravenous injection of free DIR, EL@DIR, and AS16‐EL@DIR. Data are means ± SD, *n* = 3; ^**^ *p* < 0. 01, ^***^ *p* < 0.001.

In recent years, a large number of in vitro and in vivo studies have demonstrated the unique ability of EVs to target specific organs.^[^ ^41^, ^42^, ^43^ ^]^ EVs can thus overcome natural barriers and promote the uptake, intracellular trafficking, and subsequent delivery of their contents to parent cells through endogenous mechanisms.^[^ ^44^, ^45^, ^46^, ^47^, ^48^ ^]^ To systematically evaluate the homologous targeting properties of AS16‐EL@MPLA/p‐FX, we analyzed its uptake in MC38, B16, and 4T1 tumor cells, as well as in cDC1 using CLSM and flow cytometry. Results showed that AS16‐EL@MPLA/p‐FX exhibited the highest uptake efficiency in MC38 cells, with a fluorescence intensity 5–8 times higher than that in 4T1 and B16 cells. Meanwhile, although its uptake level in cDC1 cells was lower than that in MC38 cells, it remained significantly higher than in other non‐homologous tumor cells (**Figure** **4**a–c), suggesting that the carrier has affinity for both homologous tumor cells and antigen‐presenting cells. Furthermore, we assessed the cellular‐level distribution of AS16‐EL@MPLA/p‐FX within tumor tissues in an MC38 tumor‐bearing model. Flow cytometry analysis revealed that at 24 h post‐injection, the carrier was effectively internalized by tumor cells (CD45^−^) and cDC1 cells (CD45⁺CD11c⁺XCR1⁺) in the tumor microenvironment, with uptake efficiencies of 29.6% in tumor cells and 19.2% in cDC1 cells (Figure 4d,e). These findings confirm that AS16‐EL@MPLA/p‐FX possesses the capability for dual delivery to both tumor cells and key immune cells, laying a critical foundation for subsequently triggering antitumor immune responses.

The in vivo tumor‐targeting capability of AS16‐EL@MPLA/p‐FX was subsequently investigated in an MC38 subcutaneous tumor‐bearing mouse model. Following the intravenous administration of free DIR, DIR‐labeled EL@DIR, or DIR‐labeled AS16‐EL@DIR, the distribution of DIR fluorescence was continuously monitored. At 0.5, 2, 8, 24, 48, and 72 h post‐injection, anesthetized mice were subjected to in vivo imaging using a small animal imaging system to observe the fluorescence distribution across various organs. Nanoparticle accumulation at the tumor site was readily detectable within 2 h post‐injection, and the fluorescence signal intensified in a time‐dependent manner (Figure 4f). Ex vivo organ imaging at 72 h revealed that both the non‐AS16‐modified EL@DIR group and the AS16‐EL@DIR group exhibited significantly stronger fluorescence intensity in tumor tissues compared to the free DIR group, with no notable difference observed between the two nanoparticle groups (Figure 4g,h). These results demonstrate the excellent tumor‐targeting capability of the nanoplatform and indicate that AS16 peptide modification does not compromise its homologous targeting efficiency. Although some nanoparticle accumulation was also observed in the liver, H&E staining results indicated no significant toxicity in major organs (Figure S6, Supporting Information).

### 2.6 Antitumor Efficacy of AS16‐EL@MPLA/p‐FX In Vivo

**Figure 5:**
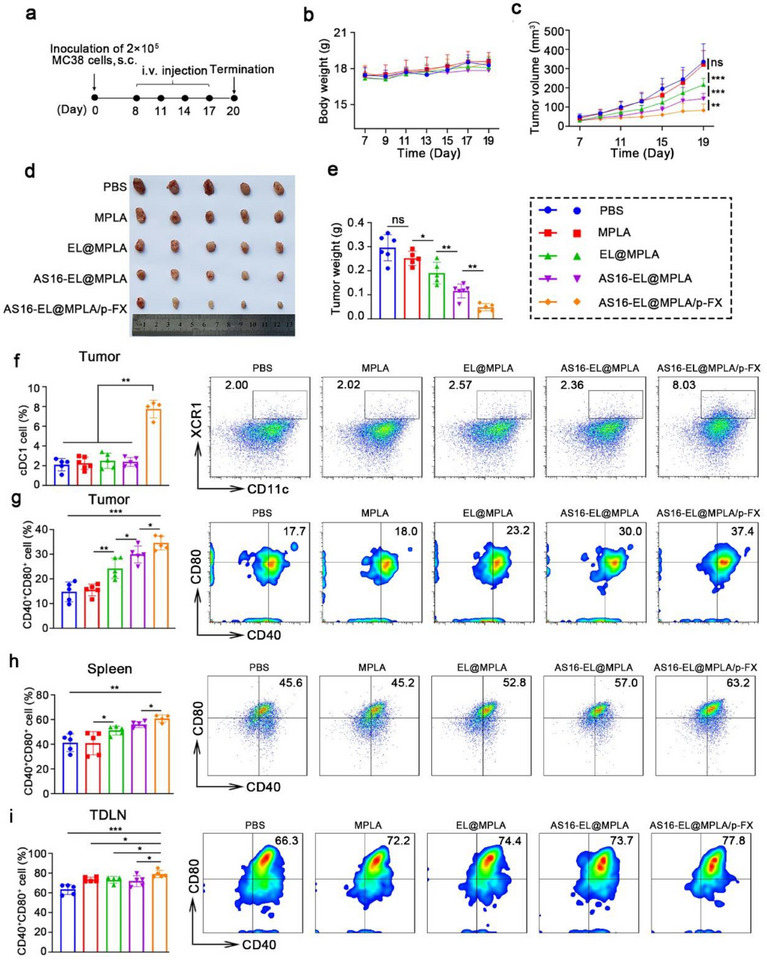
Antitumor efficacy of AS16‐EL@MPLA/p‐FX in vivo. a) Schematic illustration of AS16‐EL@MPLA/p‐FX treatment in the MC38 tumor model. b) Body weight changes of mice during treatment. c) Tumor growth curves in different groups. d) Representative tumor images and e) corresponding tumor weights. f) Flow cytometric analysis of intratumoral cDC1 populations. g) Expression of CD40 and CD80 on tumor‐infiltrating cDC1s. h,i) CD40 and CD80 expression on cDC1s in spleens and draining lymph nodes. Data are presented as mean ± SD (*n* = 5). Statistical significance: ^*^ *p* < 0.05, ^**^ *p* < 0.01, ^***^ *p* < 0.001.

To confirm that AS16‐EL@MPLA/p‐FX inhibits tumor growth, MC38 tumor‐bearing mice were established. Tumor‐bearing MC38 mice were randomly assigned to five groups and treated with either PBS, MPLA, EL@MPLA, AS16‐EL@MPLA, or AS16‐EL@MPLA/p‐FX. After the average tumor volume reached ≈50 mm^3^, the mice were intravenously administered the different formulations once every 3 days (**Figure** **5**a).

The experimental results demonstrated that the tumor volume in the free MPLA group did not exhibit significant differences compared to the PBS control group. However, administration of EL@MPLA led to a remarkable inhibition of tumor growth, with a tumor inhibition rate of 35.6%. Furthermore, in the AS16 peptide–modified AS16‐EL@MPLA group, the antitumor effect was significantly enhanced, with the tumor inhibition rate increasing to 57%. Notably, the AS16‐EL@MPLA/p‐FX group exhibited the strongest antitumor effect, achieving a tumor inhibition rate of 75.1% (Figure 5c–e and Figure S7, Supporting Information).

During the treatment period, there were no significant differences in the body weights of mice from all groups, indicating the excellent biosafety of the fused EVs prepared by us (Figure 5b). To further evaluate the potential toxicity and side effects of the fused EVs on mouse organs, histopathological analyses were performed on the major organs (heart, liver, spleen, lung, and kidney) of mice after treatment. Through H&E staining, we observed that the main organs of the treated mice did not exhibit any significant structural abnormalities compared to the PBS group, further demonstrating the high biosafety of these materials (Figure S6, Supporting Information).

### 2.7 Immune Response to AS16‐EL@MPLA/p‐FX In Vivo

During in vitro targeting experiments, we observed that fluorescently labeled AS16‐EL@MPLA/p‐FX accumulated faster and in greater quantities at tumor sites compared to free fluorescein. This phenomenon can be explained by the inherent properties of EVs, which contain membrane proteins and lipid components similar to those of the parental tumor cells, enabling them to naturally target the parental tumor cells, thus serving as an ideal drug carrier. In vitro cellular experiments have validated that p‐FX can recruit cDC1 through the expression of FLT3L and XCL1, and increase the number of cDC1 by promoting the differentiation of cDC1 precursor cells (Figure 3b). Moreover, in a co‐culture system with CD8^+^ T cells, cDC1 treated with AS16‐EL@MPLA/p‐FX significantly stimulated the proliferation of CD8^+^ T cells and the secretion of IFN‐γ (Figures S4 and S5, Supporting Information).

To investigate whether AS16‐EL@MPLA/p‐FX can recruit and activate cDC1 in tumor tissues, we performed an in vivo experiment and examined the number and maturity of cDC1 in tumor tissues after systemic administration. By preparing single‐cell suspensions from tumor tissues and performing flow cytometric analysis, we found that the number of cDC1 in the tumor did not increase in the three groups without plasmid addition (MPLA, EL@MPLA, and AS16‐EL@MPLA). However, in the full‐loaded group with plasmid addition (AS16‐EL@MPLA/p‐FX), the number of cDC1 in the tumor significantly increased, reaching 8.03% (Figure 5f).

Furthermore, we evaluated the maturation of cDC1 in tumors. By measuring the expression of maturation markers CD40 and CD80 on cDC1, we found that the MPLA group alone did not differ in cDC1 maturity compared to the PBS group (Figure 5g), likely due to the small dose of MPLA and its inability to specifically deliver to the tumor site. However, the EL@MPLA group, with the addition of the carrier, significantly stimulated the maturation of cDC1, reaching 23.2%. Given the abundance of VEGF in the tumor microenvironment, which inhibits the function of cDC1, we hypothesized that adding the AS16 peptide to the carrier, which can relieve VEGF inhibition of cDC1, would further promote cDC1 maturation. As expected, the AS16‐EL@MPLA group achieved a cDC1 maturity rate of 30%. Finally, the AS16‐EL@MPLA/p‐FX treatment group exhibited the highest cDC1 maturity rate, reaching 37.4%.

In the antitumor immune response, CD8^+^ T cells serve as the primary killer cells. Therefore, we conducted an in‐depth investigation into the specific effects of fused EV‐treated cDC1 on the number and function of CD8^+^ T cells. We found that compared to the PBS control group (14.6%), the proportion of CD8⁺ T cells in the MPLA group showed only a slight increase (16.4%), which may be due to the lack of a primary signal stimulus produced by antigen stimulation. However, when fused EVs were introduced as carriers, they provided antigen stimulation for CD8^+^ T cells, significantly enhancing their proportion (23.4%). After adding the AS16 peptide, CD8^+^ T cell proportion was restored (31.1%), indicating that the AS16 peptide effectively relieved the inhibition of VEGF on cDC1. More importantly, in the group with plasmid p‐FX added, the CD8^+^ T cells proportion reached 41.0%, which was attributed to the enhancing effect of growth factor FLT3L on cDC1 function (Figure S8, Supporting Information). Meanwhile, the IFN‐γ secretion levels of CD8⁺ T cells exhibited a consistent increasing trend (Figure S9, Supporting Information), further confirming the significant role of fused EV‐treated cDC1 in the antitumor immune response.

To further evaluate whether AS16‐EL@MPLA/p‐FX induces a systemic immune response, the maturation of cDC1s and the number of CD8^+^ IFN‐γ^+^ T cells were determined in the tumor‐draining lymph nodes and spleens. AS16‐EL@MPLA/p‐FX significantly enhanced the maturation of cDC1s (Figure 5h,i) and the IFN‐γ secretion by CD8^+^ T cells (Figures S10 and S11, Supporting Information).

To confirm the universality of AS16‐EL@MPLA/p‐FX in inhibiting tumor growth, 4T1 tumor‐bearing mouse and CT26‐tumor‐bearing mouse were established. Consistent with the results observed in MC38 tumor‐bearing mouse models, the free MPLA group did not exhibit significant differences compared to the PBS control group, whereas AS16‐EL@MPLA demonstrated marked tumor suppression. Notably, AS16‐EL@MPLA/p‐FX exhibited the most potent antitumor effect among all treatment groups (Figures S12c–e and S14, Supporting Information). Meanwhile, throughout the treatment period, body weight remained consistent across all experimental groups with no significant differences observed (Figure S12b, Supporting Information), indicating the biocompatibility of prepared fused EVs.

The maturity of DC in tumor tissues plays a crucial role in tumor killing. We further evaluated the maturity of DC in tumor tissues in 4T1 tumor‐bearing mice. The maturity of DC was evaluated by detecting the expression levels of CD80 and CD86 in DC. The results demonstrated that EL@MPLA/p‐FX could significantly stimulate the maturation of DC at the tumor site in mice, with a maturation rate of up to 46.1% (Figure S12f, Supporting Information). Among all DC subsets, the cDC1 subset is uniquely equipped with the ability to robustly cross‐present tumor antigens and subsequently activate cytotoxic CD8⁺ T cells. The results revealed that the proportion of cDC1 in the 4T1 tumor could reach 11.5% (Figure S12g, Supporting Information), which was significantly higher than that in the PBS group (where the proportion of cDC1 was 2.99%). The above research results confirm that AS16‐EL@MPLA/p‐FX can successfully recruit and activate cDC1 in the tumor tissues of 4T1 tumor‐bearing mice while promoting DC maturation.

To further evaluate whether EL@MPLA/p‐FX can induce a systemic immune response in both 4T1 and CT26 tumor‐bearing mice, we measured the number of CD8^+^ IFN‐γ^+^ T cells in the tumor‐draining lymph nodes and spleens. The results showed that in both 4T1 and CT26 tumor‐bearing mice, EL@MPLA/p‐FX effectively promote the secretion of IFN‐γ by CD8^+^ T cells (Figures S12h,i, S15, and S16, Supporting Information), thereby further enhancing the tumor‐killing ability.

To investigate the transcriptional changes induced by in situ cytokine reprogramming, we performed RNA‐seq on tumor tissues collected after treatment with AS16‐EL@MPLA/p‐FX. Transcriptomic profiling revealed a total of 705 differentially expressed genes (DEGs) between the treatment and control groups, comprising 292 upregulated and 413 downregulated genes, as visualized in the volcano plot (Figure S13a, Supporting Information). Gene Ontology (GO) enrichment analysis revealed a marked upregulation of immune‐related biological processes. Notably, the top‐enriched pathways included cytokine‐mediated signaling pathway, cellular response to cytokine stimulus, and positive regulation of cytokine and chemokine production, reflecting the activation of XCL1 and FLT3L signaling axes. In addition, pathways associated with leukocyte migration, chemotaxis, and regulation of leukocyte trafficking were significantly enriched, indicating enhanced immune cell recruitment into the tumor microenvironment. The enrichment of response to lipopolysaccharide and TLR4‐related signaling pathways further corroborated the local activation of cDC1s mediated by MPLA. Importantly, upregulation of processes related to type I interferon signaling and positive regulation of the immune system process suggest a broad activation of innate and adaptive immunity. Collectively, these findings demonstrate that AS16‐EL@MPLA/p‐FX treatment reshapes the immunosuppressive tumor microenvironment into an immune‐permissive niche by orchestrating the recruitment, differentiation, and activation of cDC1s through multifaceted immunomodulatory pathways (Figure S13b, Supporting Information). Consistently, KEGG pathway analysis further demonstrated significant enrichment of immune activation and tumor microenvironment effectively recruits and activatesremodeling pathways. Among these, cytokine–cytokine receptor interaction, IL‐17 signaling pathway, and PI3K–Akt signaling pathway were prominently upregulated, indicating potent cytokine‐driven immune activation and survival signaling. Pathways associated with immune cell adhesion and infiltration, such as focal adhesion and ECM–receptor interaction, were also enriched, aligning with the observed recruitment of cDC1s into the tumor microenvironment. Additional enriched pathways, including viral protein interaction with cytokine and cytokine receptor, relaxin signaling pathway, and neuroactive ligand‐receptor interaction, reflected broad engagement of inflammatory and tissue‐remodeling responses (Figure S13c, Supporting Information). To directly validate the activation of dendritic cell recruitment and maturation programs, we next performed hierarchical clustering of representative genes involved in DC chemotaxis and activation (Figure S13d, Supporting Information). Consistent with the KEGG enrichment results, key chemokines and cytokines exhibited robust upregulation in the treatment group, confirming that AS16‐EL@MPLA/p‐FX therapy effectively initiated transcriptional programs favoring DC infiltration and functional activation within the tumor microenvironment.

### 2.8 Antitumor Efficacy of AS16‐EL@MPLA/p‐FX Combined with Radiotherapy (RT)

**Figure 6:**
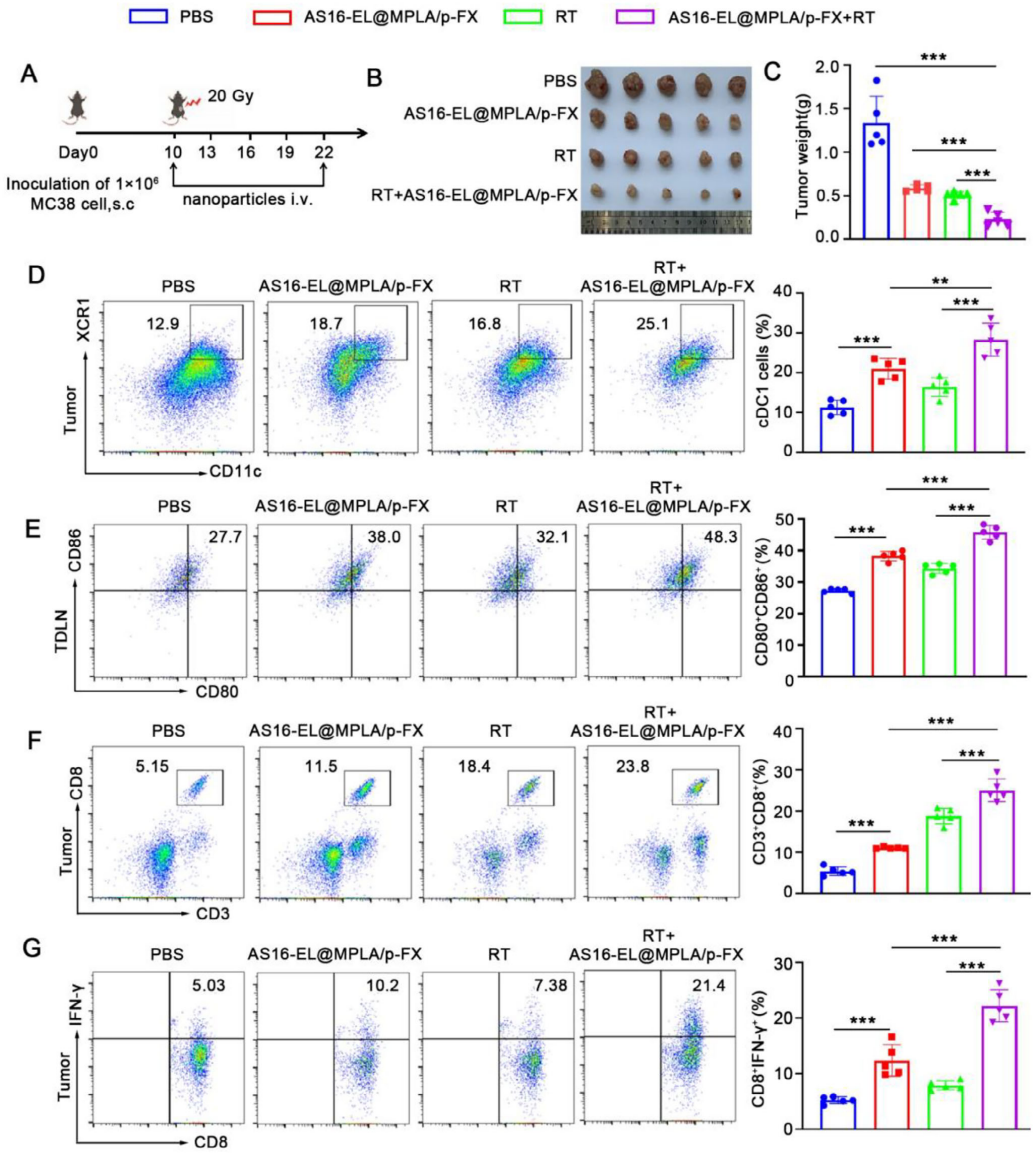
Combined RT and nanoparticle treatment inhibited tumor growth. a) Schematic illustration of AS16‐EL@MPLA/p‐FX administration in the MC38 tumor model. b) Representative tumor images and c) corresponding tumor weights in different groups. d) Flow cytometric analysis of intratumoral cDC1 populations. e) Flow cytometric assessment of tumor‐draining lymph (DLN) nodes stained with CD45, CD11c, CD80, and CD86, with corresponding quantification. f) Percentages of CD8⁺ T cells in tumors from different groups. g) Frequencies of IFN‐γ⁺ CD8⁺ T cells in tumors. Data are presented as mean ± SD (*n* = 5). Statistical significance: ^*^ *p* < 0.05, ^**^ *p* < 0.01, ^***^ *p* < 0.001.

Following our previous experimental validation, we have confirmed that AS16‐EL@MPLA/p‐FX effectively recruits and activates the key immune cell population. This activation process not only triggers a robust anti‐tumor immune response but also lays a solid foundation for subsequent treatment. Apart from directly and efficiently killing tumor cells, RT is also capable of releasing tumor‐specific antigens. These antigens serve as “antigenic markers” that facilitate the immune system to more readily recognize and attack tumor cells, thus greatly enhancing the anti‐tumor immune response.^[^ ^49^ ^]^ Therefore, we evaluated the antitumor efficacy of AS16‐EL@MPLA/p‐FX combined with RT. MC38 tumor‐bearing mice were treated with PBS, AS16‐EL@MPLA/p‐FX, RT, or their combination (RT+AS16‐EL@MPLA/p‐FX), and the tumor volumes were measured (**Figure** **6**a). On day 25, the tumors were removed, weighed, and photographed. As shown in Figure 6b,c, mice treated with AS16‐EL@MPLA/p‐FX or RT alone showed marginal inhibition of tumor growth compared with the PBS group, whereas the mice treated with AS16‐EL@MPLA/p‐FX combined with RT exhibited the strongest tumor growth inhibition. To determine whether RT+AS16‐EL@MPLA/p‐FX could recruit and activate cDC1s in vivo, the proportions of cDC1s in tumor tissues were determined using flow cytometry. As shown in Figure 6d, mice treated with RT+AS16‐EL@MPLA/p‐FX showed a marked increase in the cDC1 population (28.32%) compared with those in the other groups. Compared to the AS16‐EL@MPLA/p‐FX group and RT, RT+AS16‐EL@MPLA/p‐FX (45.78%) significantly promoted DCs maturation in vivo (Figure 6e). The intratumor CD8^+^ T cell infiltration and the function of CD8^+^ T cells were analyzed by flow cytometry. It was found that the percentages of CD8^+^ T cells and IFN‐γ‐producing CD8^+^ T cells in the RT+AS16‐EL@MPLA/p‐FX group were dramatically elevated compared with those in the RT and AS16‐EL@MPLA/p‐FX groups (Figure 6f,g; Figures S18 and S19, Supporting Information).

Based on the experimental results, we observed significant therapeutic efficacy in tumor treatment using the combined application of AS16‐EL@MPLA/p‐FX and RT. Once the AS16‐EL@MPLA/p‐FX effectively activates cDC1, these activated immune cells precisely present antigens to T cells, particularly CD8^+^ T cells, thereby initiating an efficient adaptive immune response. Subsequently, the activated CD8^+^ T cells specifically recognize and eliminate tumor cells, completing the immune cycle. This achievement brings new therapeutic strategies and hope to the field of tumor treatment.

### 2.9 Inhibition of Lung Metastasis and Abscopal Tumor Growth by AS16‐EL@MPLA/p‐FX

In previous experiment**s,** AS16‐EL@MPLA/p‐FX elicited a potent anti‐tumor immune response, highlighting its potential as a promising therapeutic approach. To further evaluate its clinical applicability and long‐term efficacy, we performed lung metastasis and tumor rechallenge experiments. The lung metastasis model was established to simulate the natural dissemination of tumors to the lungs in vivo, thereby assessing the therapeutic effectiveness of this strategy in preventing pulmonary tumor metastasis. Simultaneously, the rechallenge experiment was designed to determine whether the immune system retains its ability to recognize and eliminate tumor cells following the initial treatment, and whether the induced immune memory is sufficient to counteract a secondary tumor challenge.

**Figure 7:**
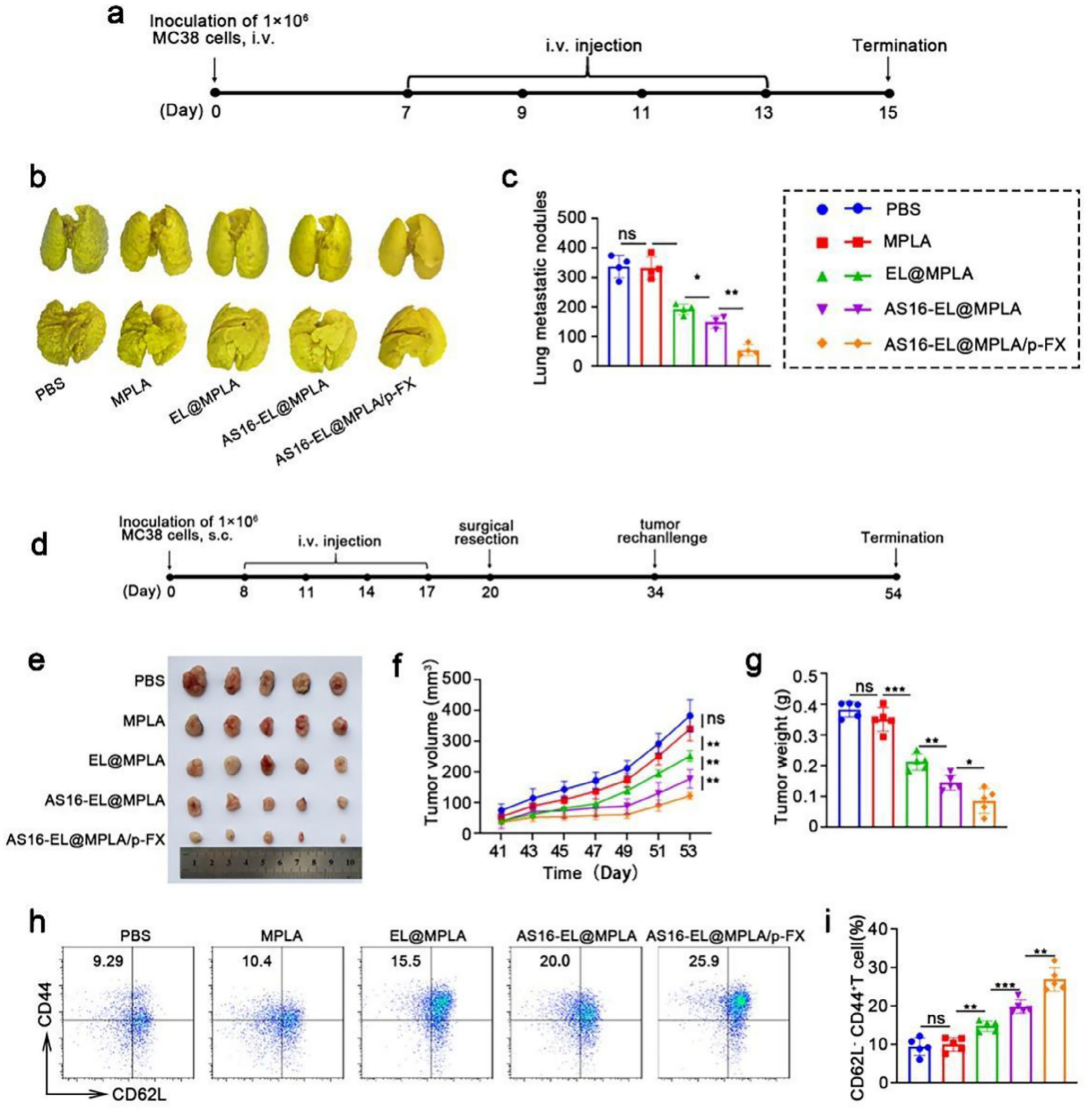
Effects of AS16‐EL@MPLA/p‐FX on lung metastasis and tumor rechallenge in the MC38 model. a) Schematic of the lung metastasis experiment. MC38 cells were injected intravenously to establish the metastasis model. After four administrations, mice were sacrificed, and lungs were collected for metastatic nodule counting. b) Representative images of pulmonary metastatic nodules. c) Quantification of pulmonary metastatic nodules (*n* = 4). d) Schematic of the tumor rechallenge experiment. MC38 tumor‐bearing mice were treated with four doses of AS16‐EL@MPLA/p‐FX, followed by surgical removal of primary tumors. Two weeks later, mice were rechallenged with MC38 cells, and tumor growth was monitored for 20 days. Spleens were harvested at the endpoint for immune analysis. e) Representative image of rechallenged tumors. f) Tumor growth curves of rechallenged tumors. g) Final tumor weights across groups. h,i) Proportions of splenic memory T cells assessed by flow cytometry. Data are presented as mean ± SD (*n* = 5). Statistical significance: ^*^ *p* < 0.05, ^**^ *p* < 0.01, ^***^ *p* < 0.001; *ns*, not significant.

We subsequently investigated the anti‐metastatic efficacy of AS16‐EL@MPLA/p‐FX in a murine model of lung metastasis. MC38 tumor cells were intravenously injected into C57BL/6 mice on day 0, followed by treatment with PBS, MPLA, EL@MPLA, AS16‐EL@MPLA, or AS16‐EL@MPLA/p‐FX on day 7 (**Figure** **7**a). On day 15 post‐tumor inoculation, the mice were sacrificed, and their lungs were harvested for evaluation of metastatic nodules. As shown in Figure 7b,c, mice treated with MPLA, EL@MPLA, or AS16‐EL@MPLA exhibited extensive lung metastases. In contrast, treatment with AS16‐EL@MPLA/p‐FX markedly suppressed tumor formation, with only a few negligible metastatic nodules observed.

We then examined whether AS16‐EL@MPLA/p‐FX could induce a durable immune memory response capable of preventing tumor recurrence. C57BL/6 mice bearing MC38 tumors received the indicated treatments and were subsequently rechallenged with MC38 tumor cells 30 days after the initial tumor inoculation (Figure 7d). Mice treated with MPLA, EL@MPLA, or AS16‐EL@MPLA did not show significant tumor suppression upon rechallenge when compared to the PBS control. Strikingly, tumor growth was significantly inhibited in mice treated with AS16‐EL@MPLA/p‐FX (Figure 7e–g; Figure S17, Supporting Information).

To elucidate the underlying mechanisms responsible for tumor suppression, we analyzed effector memory T cells in the spleens via flow cytometry. The proportion of effector memory T cells (CD3⁺CD8⁺CD44⁺CD62L^−^) in the AS16‐EL@MPLA/p‐FX‐treated group dramatically increased to 25.9%, representing a 2.8‐fold elevation compared to the PBS control group (Figure 7h,i). These findings demonstrate that AS16‐EL@MPLA/p‐FX induces a robust anti‐tumor immune response, effectively suppressing both lung metastasis and tumor recurrence. Collectively, these results provide compelling evidence supporting the anti‐metastatic and immune memory‐enhancing capabilities of AS16‐EL@MPLA/p‐FX. These outcomes offer critical insights into its clinical translational potential and therapeutic efficacy in real‐world settings, thereby serving as a valuable reference for the rational design of future clinical trials.

## 3 Discussion

DCs are pivotal for presenting antigens and initiating antitumor immune responses. Nevertheless, their infiltration into the TME is often limited, resulting in suboptimal T cell priming. Despite the progress of DC‐centered therapeutic strategies, limited therapeutic efficacy has been achieved. A major challenge lies in the functional diversity among DC subsets, which exhibit significant differences in their capacity for antigen processing and T cell activation. Of these DC subsets, cDC1s are uniquely proficient in cross‐presenting tumor antigens and activating cytotoxic T lymphocytes. Therefore, approaches that specifically promote the recruitment and functional activation of cDC1s are essential for enhancing the therapeutic efficacy of cancer immunotherapy.

In this study, we leveraged principles of synthetic biology to reprogram tumor cells into factories capable of reshaping cDC1 function. To achieve this, we engineered a tumor‐derived EV, designated AS16‐EL@MPLA/p‐FX, which integrates cytokine‐secreting plasmid and immunostimulatory molecules to promote cDC1 recruitment and activation. After intravenous injection, AS16‐EL@MPLA/p‐FX effectively accumulates in the tumor site due to its homing ability. In the tumor microenvironment, the AS16 peptide on the EV surface dissociates under MMP‐2 cleavage, facilitating EV internalization by tumor cells. Once internalized, the plasmids expressed in tumor cells produce XCL1 and FLT3L, significantly promoting the recruitment and differentiation of cDC1. Subsequently, the released MPLA and AS16 peptide synergistically activate cDC1 and maintain its activity, triggering a persistent antitumor immune response and effectively inhibiting tumor growth, metastasis, and recurrence.

Importantly, cDC1 function is often suppressed by immunosuppressive factors in the tumor microenvironment, leading to their scarcity and impaired activity. Our developed AS16‐EL@MPLA/p‐FX nanoparticle overcomes this challenge by effectively recruiting and activating cDC1 at the tumor site, thereby amplifying antigen presentation and strengthening the CD8⁺ T cell‐mediated immune response. Experimental results demonstrate that this nanoparticle markedly increases the number of intratumoral cDC1, enhances antigen uptake and presentation, and restores cDC1 maturation even under VEGF‐driven immunosuppression. Consequently, AS16‐EL@MPLA/p‐FX significantly stimulates CD8⁺ T cell proliferation and IFN‐γ secretion, further augmenting cytotoxicity against tumor cells.

Furthermore, when combined with RT, AS16‐EL@MPLA/p‐FX exhibited superior therapeutic effects. RT not only exerts local cytotoxicity but also promotes immunogenic cell death, which synergizes with cDC1 activation to amplify systemic immunity. In lung metastasis and rechallenge models, the nanoparticle demonstrated durable antitumor effects, validating its efficacy in complex tumor settings. It not only inhibited tumor progression but also promoted cDC1 and CD8⁺ T cell infiltration into tumor sites, while simultaneously enhancing systemic immunity in the spleen and lymph nodes.

From a translational perspective, it should be noted that while Lipo 2000 was employed in this study for mechanistic validation and proof‐of‐concept demonstration, its clinical applicability is limited. Future work will focus on substituting it with clinically approved lipid nanoparticle (LNP) components, which are safer and more translatable for therapeutic applications. Incorporating such LNP systems will further strengthen the clinical relevance of AS16‐EL@MPLA/p‐FX, bridging the gap between experimental proof‐of‐concept and potential clinical translation.

In summary, the AS16‐EL@MPLA/p‐FX functionalized EV nanoparticle effectively recruits and activates cDC1, enhances CD8⁺ T cell immunity, and synergizes with RT to achieve robust tumor suppression. By gradually refining delivery systems toward clinically viable formulations, this strategy provides a promising framework for next‐generation tumor immunotherapy.

## 4 Experimental Section

### Mice and Cell Lines

Female C57BL/6 mice (6–8 weeks old) were purchased from Charles River (Beijing, China) and housed in a pathogen‐free facility at Zhengzhou University. All animal experiments were approved by the Zhengzhou University Ethics Committee in accordance with national and institutional guidelines (ZZUIRB2021‐30). MC38 cells (CVCL_B288) were obtained from the Cell Bank of the Chinese Academy of Sciences in September 2018 and were confirmed to be free of mycoplasma contamination. Cells were maintained in DMEM supplemented with 10% fetal bovine serum (FBS; BI, Israel) at 37 °C in a humidified incubator with 5% CO_2_.

### Plasmid Construction

The coding sequences (CDS) of XCL1 (NM_008510.2) and FLT3L (NM_013520.3) were cloned into the *EcoRI* and *BamHI* restriction sites of the plvx‐puro vector (BGI). The plasmid was transformed into *E. coli* DH5α cells and amplified in LB medium. Plasmid extraction and storage at −20 °C were performed according to the manufacturer's instructions (Cwbio).

### Extracellular Vesicle Isolation

When MC38 cells reached 80–90% confluency, the culture medium was replaced with fresh medium without FBS. After 48 h, the conditioned medium was collected and sequentially centrifuged at 3500 rpm for 10 min and 10 000 × g for 30 min to remove cells and cell debris. The clarified supernatant was then ultracentrifuged at 100 000 × g for 90 min at 4 °C. The pellet obtained was washed once with PBS (pH 7.4) and ultracentrifuged again at 100 000 × g for 90 min to eliminate contaminating proteins. Finally, the purified EVs were resuspended in PBS (pH 7.4) and stored at −80 °C until further use.

### Peptide Synthesis

The peptide C16‐RRKKPLGLAG‐AS16 was synthesized using standard solid‐phase Fmoc chemistry. All amino acids used in the synthesis were purchased from GL Biochem Ltd. (Shanghai, China). The synthesized peptides were purified by RP‐HPLC (Shimadzu, Japan), and their molecular weight was confirmed by electrospray ionization mass spectrometry (ESI‐MS, Waters, USA).

Cleavage of C16‐RRKKPLGLAG‐AS16 by rhMMP‐2: In assay buffer (50 mm Tris, 10 mm CaCl_2_, 150 mm NaCl, 0.05% Brij‐35, pH 7.4), C16‐RRKKPLGLAG‐AS16 (100 µm) was incubated with an equal volume of rhMMP‐2 activated by APMA (0.2 µg mL^−1^) for 3 h at 37 °C. Reaction products were analyzed by ESI‐MS.

### Preparation of AS16‐EL@MPLA/p‐FX

Lipofectamine 2000 (Lipo2000, Invitrogen) and the indicated plasmids were each diluted in DMEM, mixed, and incubated at room temperature for 5 min to form the plasmid–liposome complex. Subsequently, EVs, MPLA (Invivogen), and AS16 peptide were added to the plasmid–liposome complex and incubated at 37 °C for 12 h. The ratio of Lipo2000: EVs: plasmid: MPLA: AS16 peptide in 1 mL PBS was 20 µL: 40 µg: 10 µg: 40 ng: 4 µg. After incubation, the complex was purified by differential centrifugation to remove unencapsulated components. Finally, the re‐extracted complex was extruded through a polycarbonate porous membrane (200 nm) to obtain AS16‐EL@MPLA/p‐FX.

### Characterization of Different AS16‐EL@MPLA/p‐FX Formulations

The ζ‐potential and hydrodynamic size of AS16‐EL@MPLA/p‐FX were measured using a Zetasizer Nano ZS (Malvern, UK). The morphology of AS16‐EL@MPLA/p‐FX was observed by TEM (HT7700, Hitachi, Japan). EVs and liposomes were labeled with DID and DIO, respectively, and the fusion efficiency of AS16‐EL@MPLA/p‐FX was evaluated by CLSM. To assess protein integrity, Western blotting was performed to analyze the expression of CD9, TSG101, and GAPDH. The loading ratio and encapsulation efficiency of plasmid, MPLA, and AS16 peptide were determined using a Qubit Fluorometer (Life Technologies), LAL assay (BIOENDO), and LC‐MS (Waters, USA), respectively.

### In Vitro Cytotoxicity

The cytotoxicity of AS16‐EL@MPLA/p‐FX was evaluated using 3‐(4,5‐dimethylthiazol‐2‐yl)‐2,5‐diphenyltetrazolium bromide (MTT). MC38 cells were seeded in 96‐well plates at a density of 3000 cells per well. Cells were incubated at 37 °C in the presence of PBS, MPLA (40 ng mL^−1^), p‐FX, AS16 peptide, EL, Lipo2000, or AS16‐EL@MPLA/p‐FX for 24, 48, and 72 h. Afterward, MTT solution (5 mg mL^−1^, Solarbio, China) was added and incubated for 4 h. Following removal of the medium, the resulting formazan crystals were dissolved in DMSO (150 µL per well). Absorbance at 490 nm was measured using a SpectraMAX iD5 reader (Molecular Devices, USA).

### MC38 Transfection

When MC38 cells reached 80% confluence, they were incubated with AS16‐EL@MPLA/p‐FX (containing 40 ng mL^−1^ MPLA) in six‐well plates. After 24 h of incubation, the medium was replaced with fresh complete DMEM. Subsequently, the cells were washed three times with PBS, fixed with 4% paraformaldehyde, permeabilized using the Permeabilization Reagent (eBioscience, 00‐8333‐56), and analyzed by flow cytometry (BD FACSCelesta) following staining with anti‐mFLAG‐PE (L5, Biolegend) and anti‐mHis‐APC (J095G46, eBioscience).

### DC Recruitment

cDC1s were generated by culturing bone marrow cells flushed from the femurs of 6–8 weeks old C57BL/6 mice in medium supplemented with 200 ng mL^−1^ FLT3L (eBioscience). Half of the medium was replaced every 4 days, and non‐adherent and loosely adherent immature DCs were harvested on day 12. MC38 cells were seeded into 24‐well plates at a density of 3 × 10^5^ cells per well. On the following day, the medium was replaced with 600 µL of medium containing 10% serum and AS16‐EL@MPLA/p‐FX or other drugs, while a Transwell insert was loaded with 250 µL of cDC1 suspension (5 × 10^5^ cells per well). After 24 h, the culture medium from the bottom chamber was collected, and the cells were stained with anti‐mCD11c‐PE (N418, eBioscience) and anti‐mXCR1‐APC (ZET, Biolegend), followed by analysis using flow cytometry (BD FACSCelesta). The gating strategies for flow cytometric experiments are shown in Figure S20 (Supporting Information).

### DC Maturation

cDC1s, 5 × 10^5^, were cultured in six‐well plates with various treatments, including PBS (pH 7.2), VEGF (20 ng mL^−1^) + MPLA (40 ng mL^−1^), VEGF + EL@MPLA, VEGF + AS16‐EL@MPLA, VEGF + AS16‐EL@MPLA/p‐FX, VEGF + AS16‐EL@MPLA/p‐FX + MMP‐2, and AS16‐EL@MPLA/p‐FX. After 24 h, cDC1s were harvested and stained with anti‐mCD11c‐PE (N418, eBioscience), anti‐mCD80‐eFluor710 (16‐10A1, Biolegend), anti‐mCD40‐FITC (HM40‐3, eBioscience), anti‐mCD86‐BV421 (BU63, eBioscience) and anti‐mMHC‐II‐FITC (M5/114.15.2, eBioscience). The stained cells were subsequently analyzed by flow cytometry.

### T Cell Activation

To evaluate CD8^+^ T cell activation, IFN‐γ production, and CD8^+^ T cell proliferation were measured. Briefly, cDC1s were stimulated with various treatments for 24 h to induce the expression of CD40 and CD80. Stimulated cDC1s (5 × 10^4^ cells per well) were then co‐cultured with CD8^+^ T cells (5 × 10^4^ cells per well) isolated from C57BL/6 mice using the CD8^+^ T Cell Isolation Kit (19835A, STEMCELL).

### In Vitro Cellular Targeting

CLSM and flow cytometric assay (FCA) were employed to evaluate the targeting ability. For CLSM observation, MC38, B16, and 4T1 cells were seeded at 5 × 10^5^ cells per confocal dish. After 24 h, the medium was replaced with fresh medium containing DiO‐labeled AS16‐EL@MPLA/p‐FX (100 µg mL^−1^). Following 3 h of incubation, cells were stained with Hoechst 33342 and imaged by CLSM. For FCA, after 3 h of co‐culture according to the strategy described above, cells were digested with 2.5% trypsin, and the mean fluorescence intensity was measured by flow cytometry to quantify nanoparticle uptake.

### In Vivo Distribution

The fluorescent dye DIR was co‐assembled with nanoparticles (EL@MPLA/p‐FX and AS16‐EL@MPLA/p‐FX), while free DIR was used as a control. C57BL/6 mice were subcutaneously inoculated with 1 × 10^6^ MC38 cells in the right flank. When the average tumor volume reached 80–100 mm^3^, mice were intravenously injected via the tail vein with either free DIR or DIR‐labeled nanoparticles at a DIR dose of 0.25 mg kg^−1^. Fluorescence distribution was monitored at predetermined time points using an in vivo imaging system (IVIS Lumina III, USA). After 72 h, tumors and major organs were excised, and fluorescence intensity was quantitatively measured.

### In Vivo Anti‐Tumor Studies

MC38 cells (2 × 10^5^) were first injected subcutaneously into the right flank of C57BL/6 mice. When the average tumor volume reached ≈≈50 mm^3^, mice were randomized and intravenously (*i.v*.) treated with PBS, free MPLA (400 ng kg^−1^), EL@MPLA, AS16‐EL@MPLA, or AS16‐EL@MPLA/p‐FX once every three days. Tumor dimensions were measured using a digital caliper, and tumor volumes were calculated according to the formula: volume = 1/2 × length × width × height. To further evaluate biosafety, major organs were collected at the end of treatment and subjected to H&E staining.

In addition to the MC38 model, two additional tumor models were established using CT26 (2 × 10^5^ cells, BALB/c mice) and 4T1 (1 × 10^6^ cells, BALB/c mice) with the same treatment regimen, except for the different initial tumor cell inoculation numbers.

### Ex Vivo Assay

After treatment, tumors from all groups of mice were harvested and digested with collagenase IV and DNase I for 40 min. The resulting single‐cell suspensions were stained with flow cytometry antibodies, including anti‐mCD45‐FITC (30‐F11, eBioscience), anti‐mCD3‐eFluor710 (17A2, eBioscience), and anti‐CD8a‐PE (53‐6.7, eBioscience), to determine the proportion of CD8⁺ T cells. The activation of cDC1s was further assessed using anti‐mCD45‐PE, anti‐mCD11c‐APC, anti‐mXCR1‐BV650, anti‐mCD80‐eFluor710, and anti‐mCD40‐FITC by flow cytometry. After gentle mechanical disruption, cells isolated from tumor‐draining lymph nodes (TDLNs) and spleens were incubated with 20 ng mL^−1^ PMA (Sigma) and 1 µm ionomycin (Sigma) for 4 h in the presence of a protein transport inhibitor (555029, BD Biosciences). These cells were subsequently collected for evaluation of IFN‐γ expression using anti‐mCD3‐eFluor710, anti‐mCD8‐PE, and anti‐mIFN‐γ‐APC by flow cytometry. In addition, DC maturation in TDLNs and spleens was analyzed with anti‐mXCR1‐BV650, anti‐mCD80‐eFluor710, and anti‐mCD40‐FITC staining.

### In Vivo RT‐Combined Treatment Model

MC38 tumor cells in the logarithmic growth phase were harvested, and 1 × 10^6^ cells per mouse were subcutaneously inoculated into the right flank of C57BL/6 mice. When the average tumor volume reached ≈80–100 mm^3^, mice were randomized into four groups (PBS, AS16‐EL@MPLA/p‐FX, RT, and AS16‐EL@MPLA/p‐FX + RT) with appropriate labeling. Treatments were administered via intravenous injection through the tail vein, with the MPLA‐equivalent dose set at 400 ng kg^−1^. 4 h after the first injection, mice in the RT and AS16‐EL@MPLA/p‐FX + RT groups received localized tumor irradiation at a dose of 20 Gy. Subsequent administrations of the nanoparticles were performed once every three days for a total of five doses.

### Lung Metastasis Model

C57BL/6 mice were intravenously injected with 1 × 10^6^ MC38 cells. On day 7, the mice were randomized into groups and treated with PBS, free MPLA (400 ng kg^−1^), EL@MPLA, AS16‐EL@MPLA, or AS16‐EL@MPLA/p‐FX via tail vein injection every three days for a total of four doses (n = 4 per group). On day 15, the mice were sacrificed, and the lungs were excised for quantification of metastatic tumor nodules.

### Tumor Rechallenge

C57BL/6 mice (6–8 weeks old) were subcutaneously injected with 1×10^6^ MC38 tumor cells in the right flank. When tumors reached a volume of 80–100 mm^3^, the mice (*n* = 5 per group) were treated with PBS, MPLA (400 ng kg^−1^), EL@MPLA, AS16‐EL@MPLA, or AS16‐EL@MPLA/p‐FX via tail vein injection once every three days. On day 20, tumors were surgically removed. Subsequently, 1×10^6^ MC38 cells were injected subcutaneously into the left flank of the mice. Tumor volumes were measured every two days using a digital caliper and calculated according to the previously described formula. On day 54, tumor‐specific memory T cells were analyzed by flow cytometry.

### Statistical Analysis

For comparisons between two groups, unpaired two‐tailed Student's *t*‐tests were performed. For experiments involving multiple groups, one‐way analysis of variance (ANOVA) followed by Tukey's post hoc test was used. All values are shown as means ± SD. The sample size (*n*) for each statistical analysis is specified in the corresponding figure legends, with at least three independent biological replicates unless otherwise stated. ^*^ *p* < 0.05, ^**^ *p* < 0.01, and ^***^ *p* < 0.001 were considered statistically significant. All analyses were conducted using Microsoft Excel and GraphPad Prism (GraphPad Software, San Diego, CA, USA).

## Conflict of Interest

The authors declare no conflicts of interest.

## Supporting information



Supporting Information

## Data Availability

The data that support the findings of this study are available from the corresponding author upon reasonable request.
