# Supplementary material for: In Situ Engineering of Tumor Cells as Self‐Sustaining cDC1 Programming Factories for Effective Cancer Immunotherapy
Source: Adv Sci (Weinh). 2025 Nov 3;13(4):e08632. doi: 10.1002/advs.202508632 (PMC12822401; doi:10.1002/advs.202508632)
Supplement: Supplementary file 1 — Supporting Information [file ADVS-13-e08632-s001.docx]

In Situ Engineering of Tumor Cells as Self-Sustaining cDC1 Programming Factories for Effective Cancer Immunotherapy

Supplemental Figures


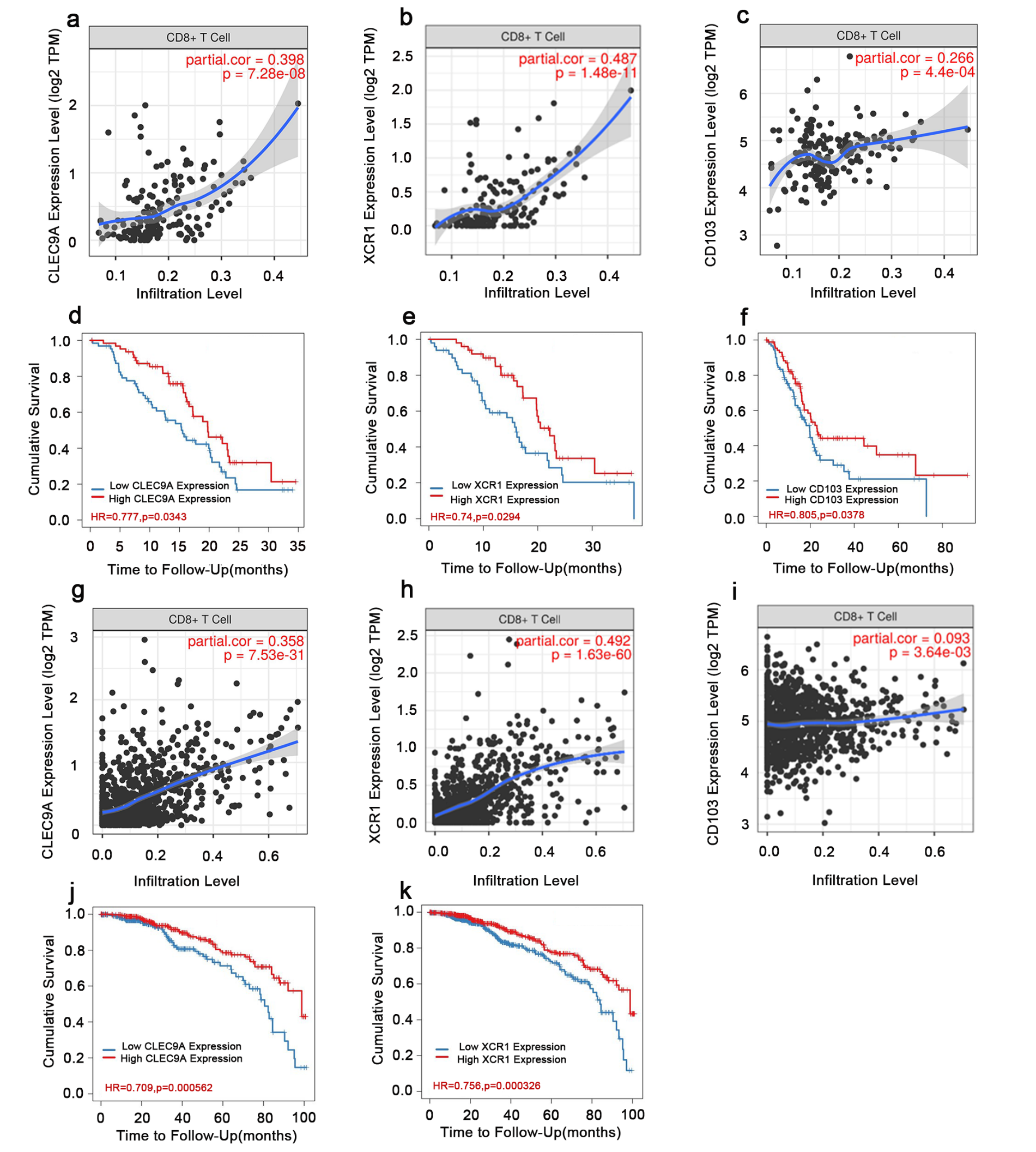


Figure S1. Association of cDC1 signature genes (CLEC9A, XCR1, and CD103) with CD8^+^ T cell infiltration and patient survival in pancreatic and breast cancers. (a-c) Correlation analysis between CLEC9A (a), XCR1 (b), and CD103 (c) expression and CD8^+^ T cell infiltration levels in pancreatic cancer.(d-f) Kaplan-Meier survival analysis of pancreatic cancer patients stratified by CLEC9A (d), XCR1 (e), and CD103 (f) expression.(g-i) Correlation analysis between CLEC9A (g), XCR1 (h), and CD103 (i) expression and CD8^+^ T cell infiltration levels in breast cancer.(j-k) Kaplan-Meier survival analysis of breast cancer patients stratified by CLEC9A (j) and XCR1 (k) expression.


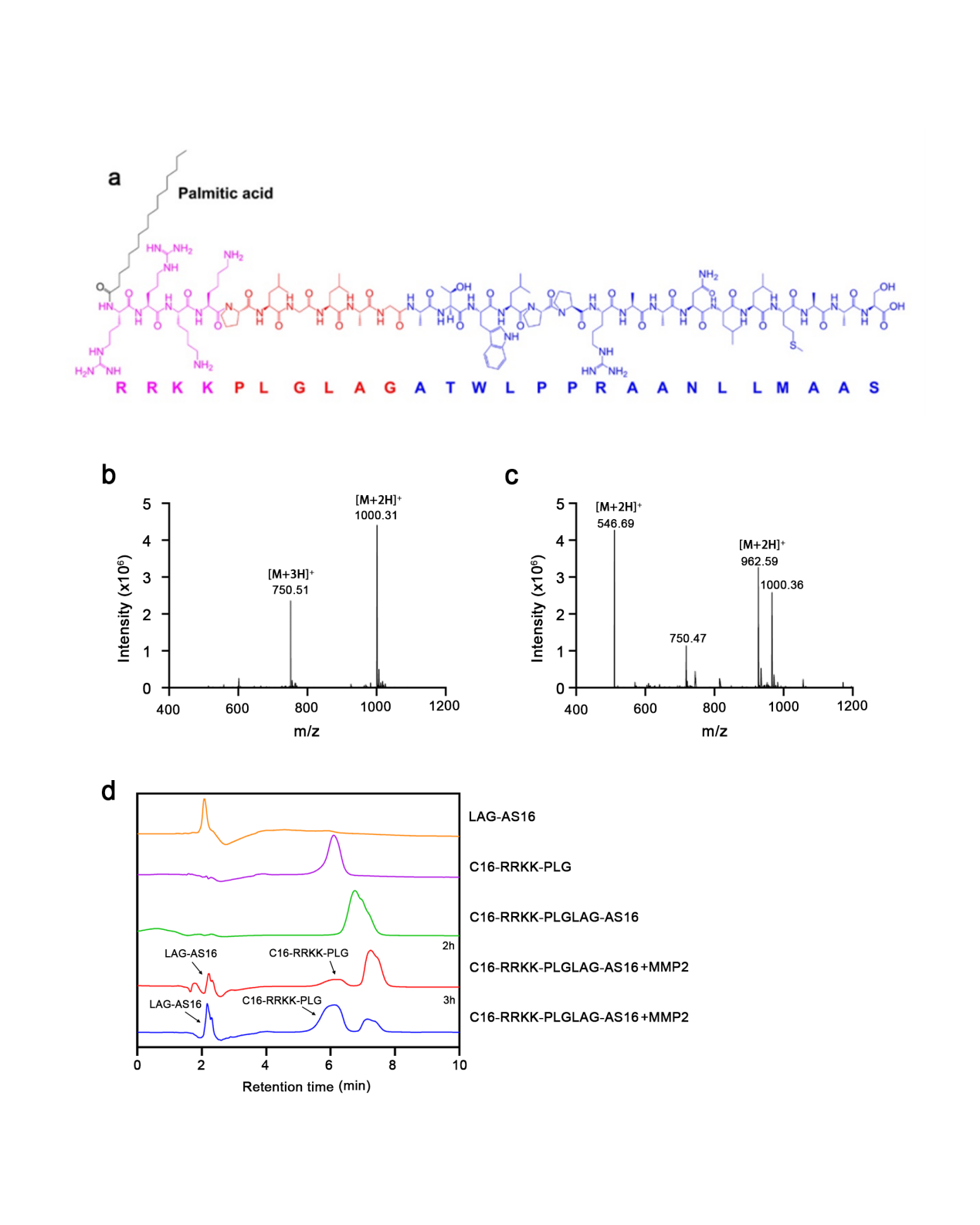


Figure S2. C16-AS16 peptide synthesis and experiment of enzyme digestion. a, Structural formula of AS16 peptide modified by palmitic acid (C16-RRKK-PLGLAG-AS16). b, Mass spectrum of C16-RRKK-PLGLAG-AS16 peptide (MW, 2998.65Da, [M+2H]^+^ 999.55Da, [M+3H]^+^749.66Da). c, C16-RRKKPLGLAG-AS16 peptide was successfully digested by MMP-2 to obtain C16-RRKKPLG (MW, 1092Da, [M+2H]^+^ 546Da) fragment and LAG-AS16 fragment (MW, 1924Da, [M+2H]^+^ 962Da). d, HPLC chromatograms of LAG-AS16, C16-RRKK-PLG, C16-RRKK-PLGLAG-AS16 and C16-RRKK-PLGLAG-AS16+MMP2.


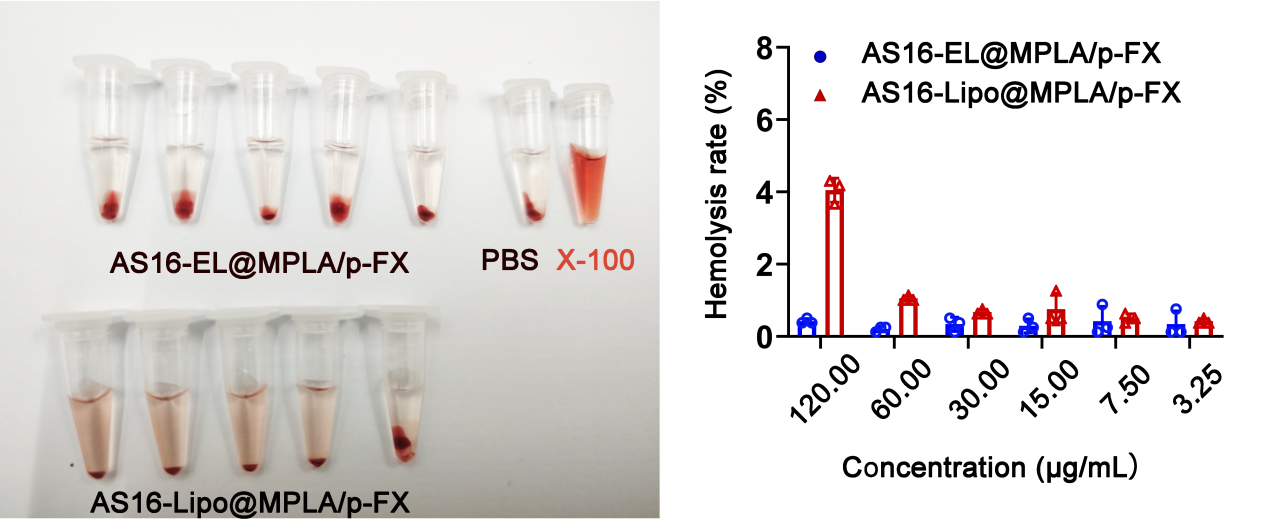


Figure S3. Hemolysis rate of AS16-EL@MPLA/p-FX and AS16-Lipo@MPLA/p-FX on sheep erythrocytes.


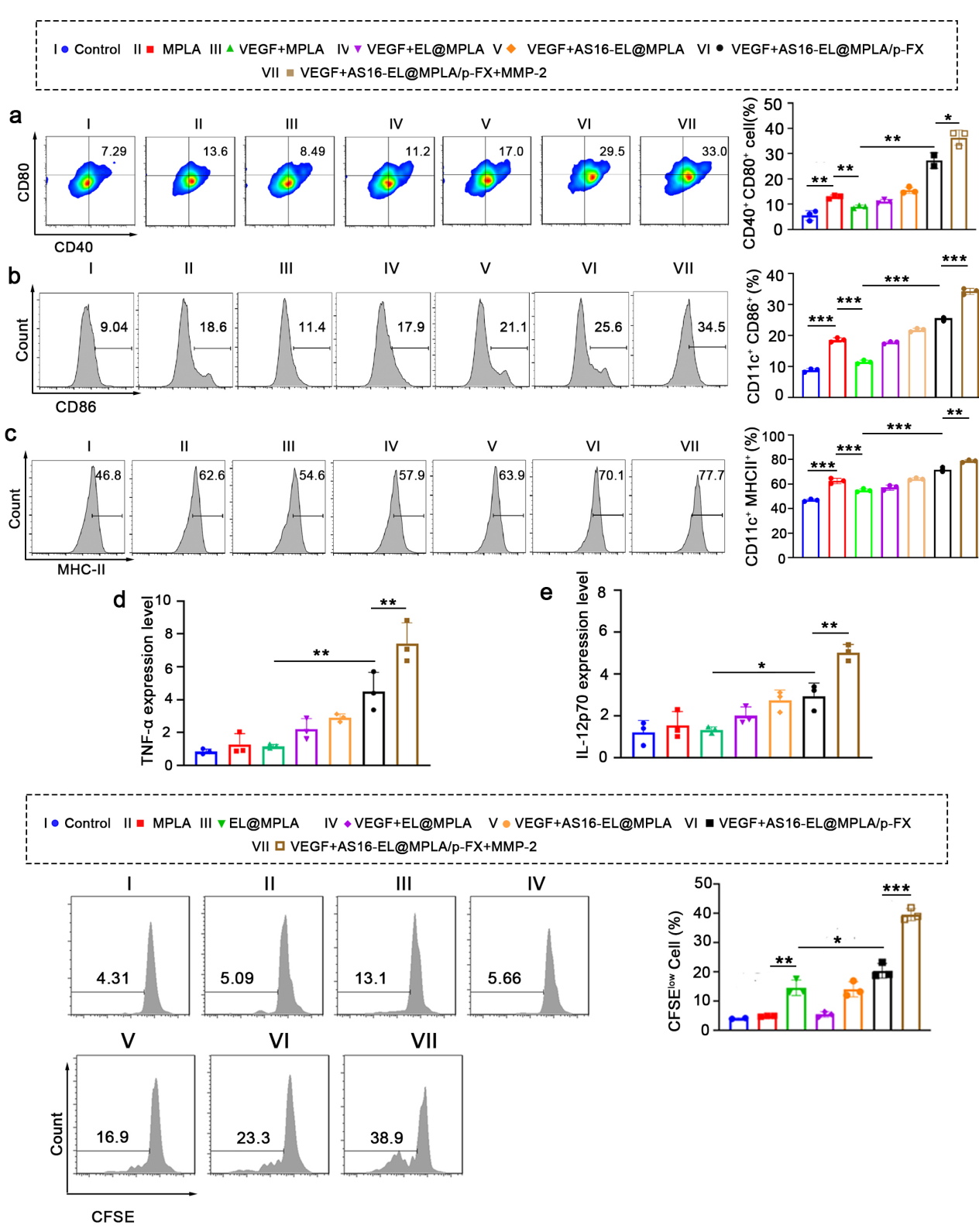


Figure S4. cDC1s were incubated with different drugs for 4 h and then cocultured with CFSE (carboxyfluorescein succinimidyl ester)-labeled CD8^+^ T cells for 72 h. The proliferation of CD8^+^ T cells was measured using flow cytometry.


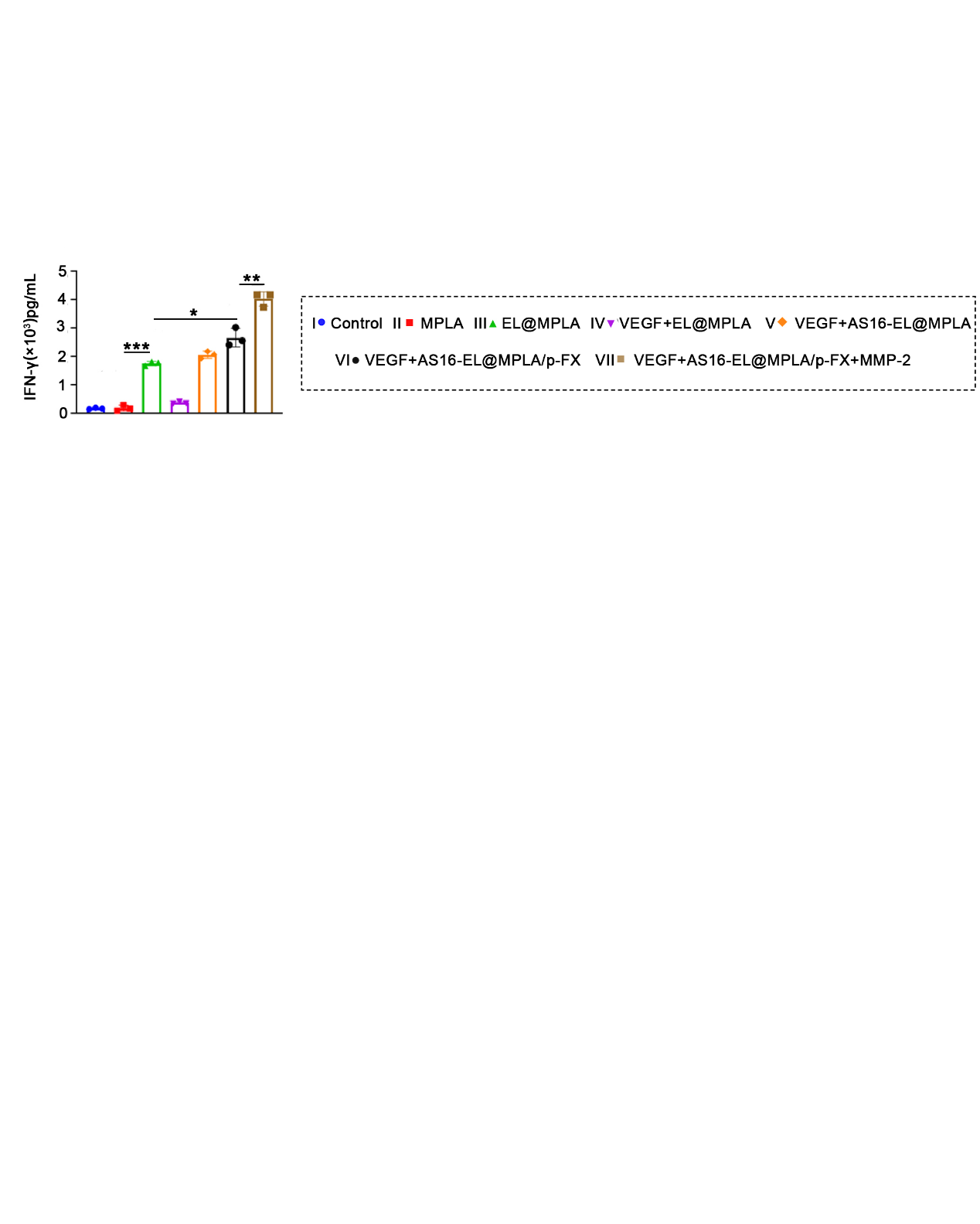


Figure S5. The amount of IFN-γ secreted by the CD8^+^ T cells was determined using an ELISA.


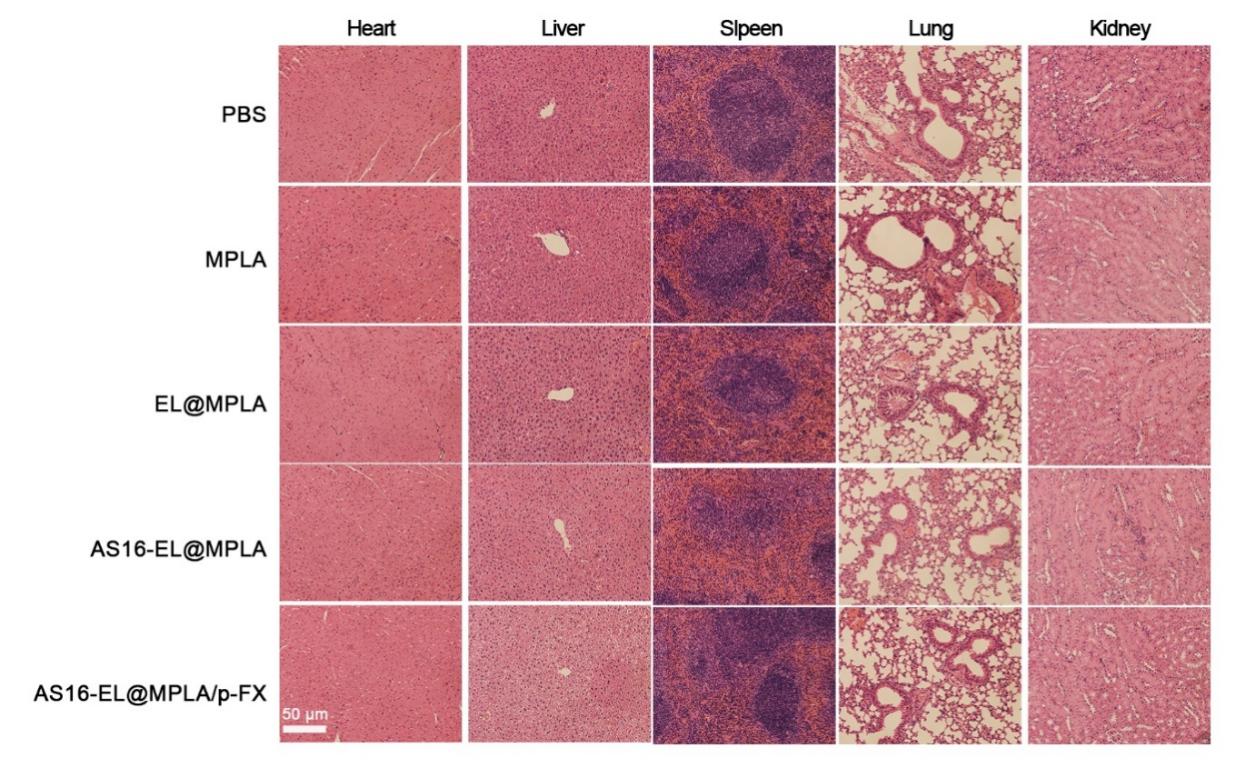


Figure S6. H&E staining of heart, liver, spleen, lung and kidney in the mice of different groups.


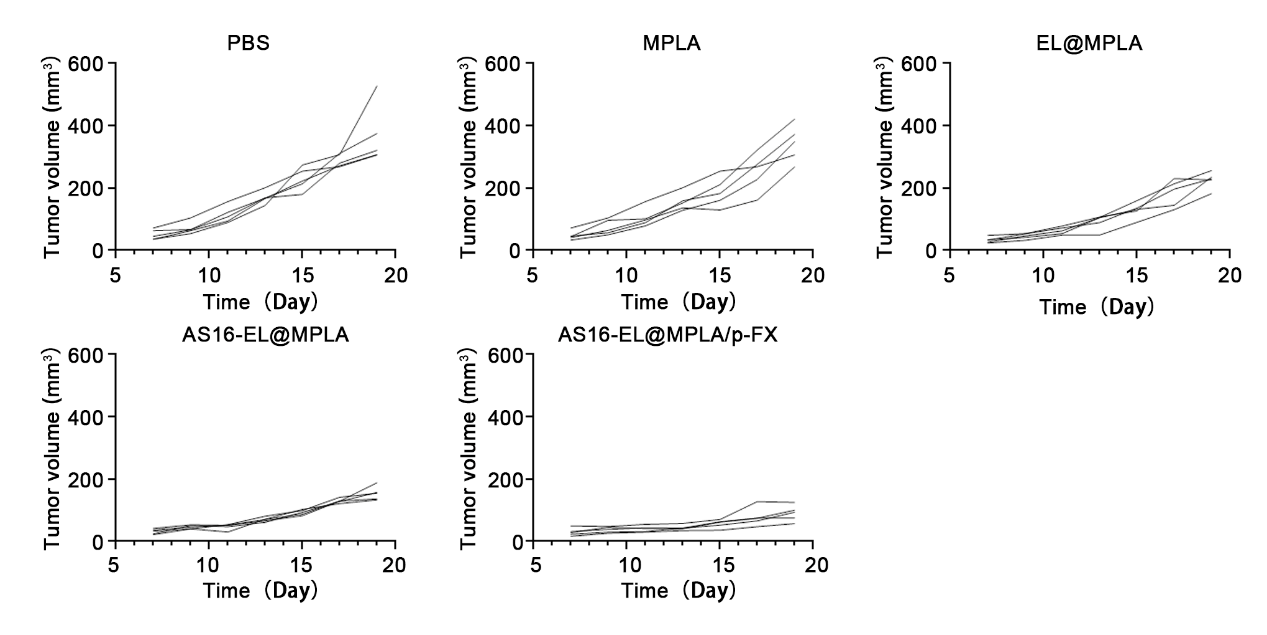


Figure S7. AS16-EL@MPLA/p-FX inhibited growth in subcutaneous MC38 model.

Each curve represents the growth of a single tumor in an individual mouse. Data are presented as the mean ± SD, *n* = 5.


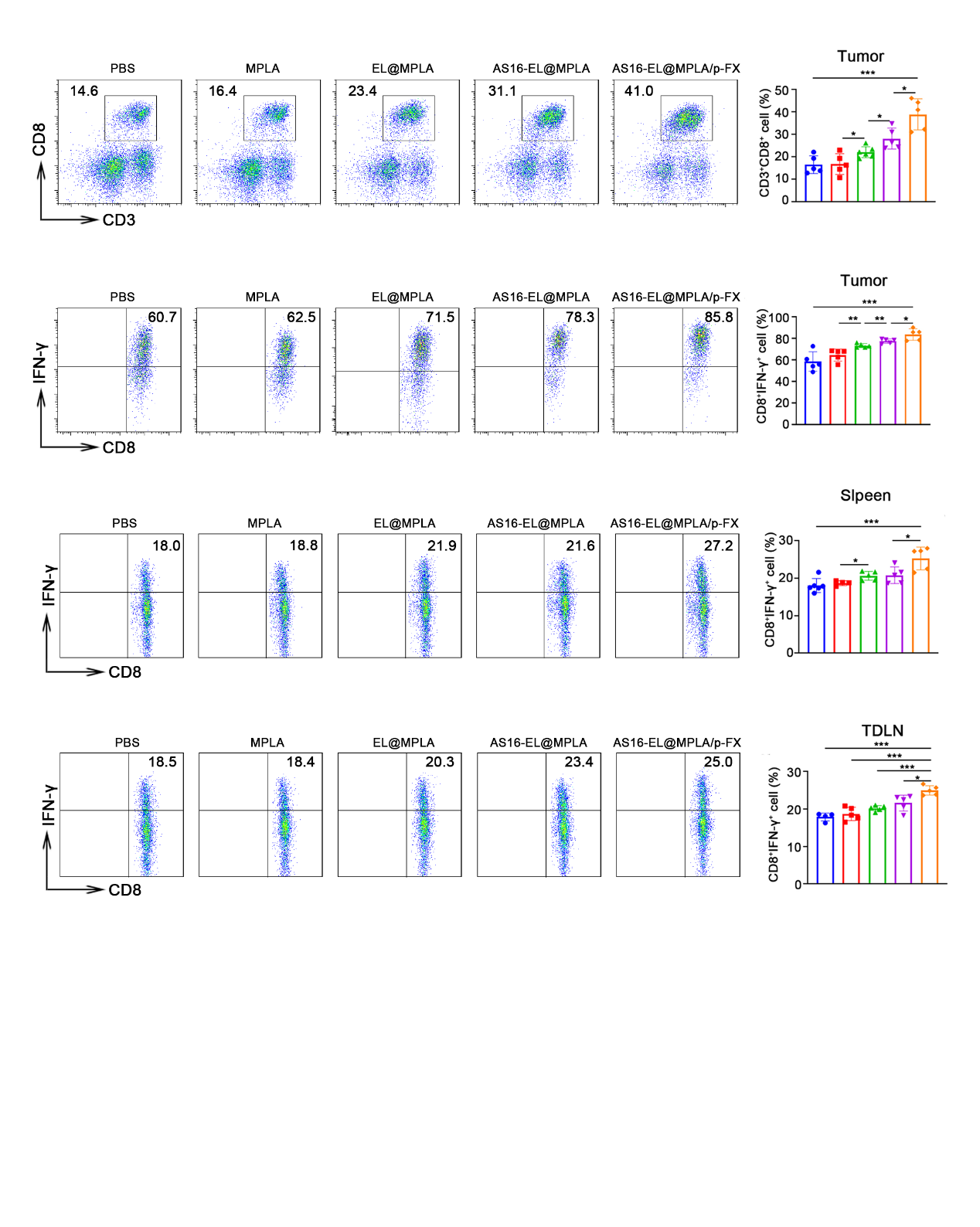


Figure S8. Representative flow plots depicting the percentages of CD8^+^ T cells in tumor. *n* = 5.


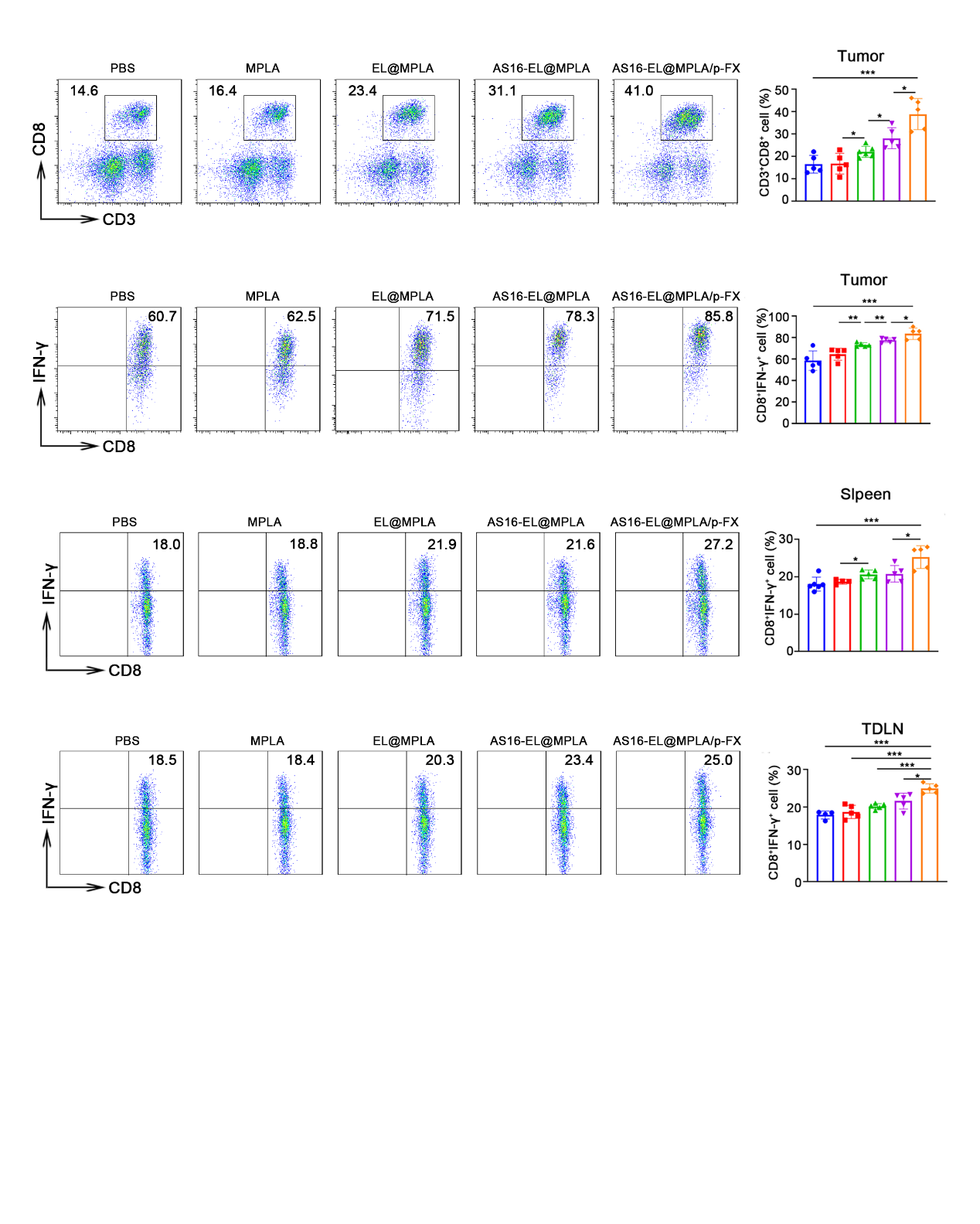


Figure S9. Representative flow plots depicting the proportion of CD8^+^ T cells expressing IFN-γ in tumor. After tumor cells were stimulated with 20 ng mL^−1^ PMA and 1 μm ionomycin, together with 1 μg mL^−1^ protein transport inhibitor for 4 h, flow cytometry was used to detect CD8^+^ T cells expressing IFN-γ. *n* = 5.


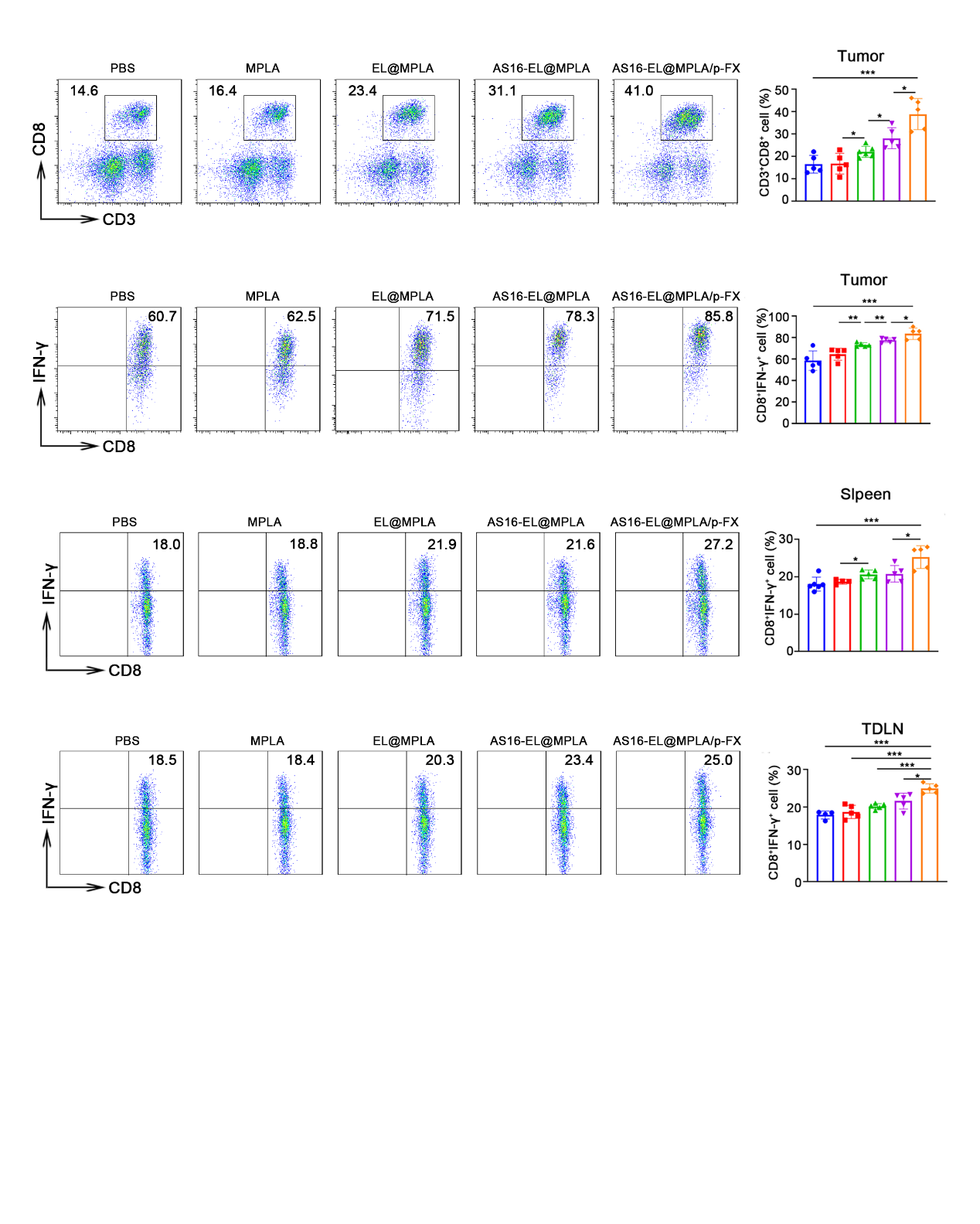


Figure S10. Representative flow plots depicting the proportion of CD8^+^ IFN-γ^+^ T cells in draining lymph nodes. *n* = 5


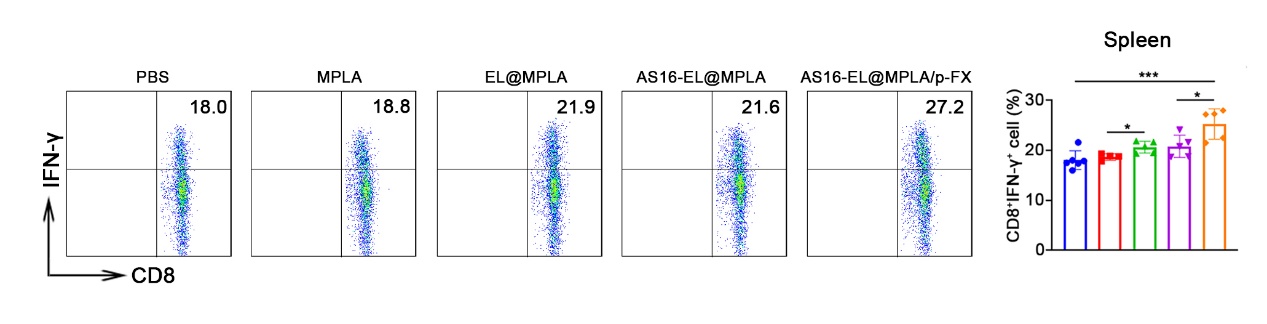


Figure S11. Representative flow plots depicting the percentages of CD8^+^ IFN-γ^+^ T cells in spleen. *n* = 5


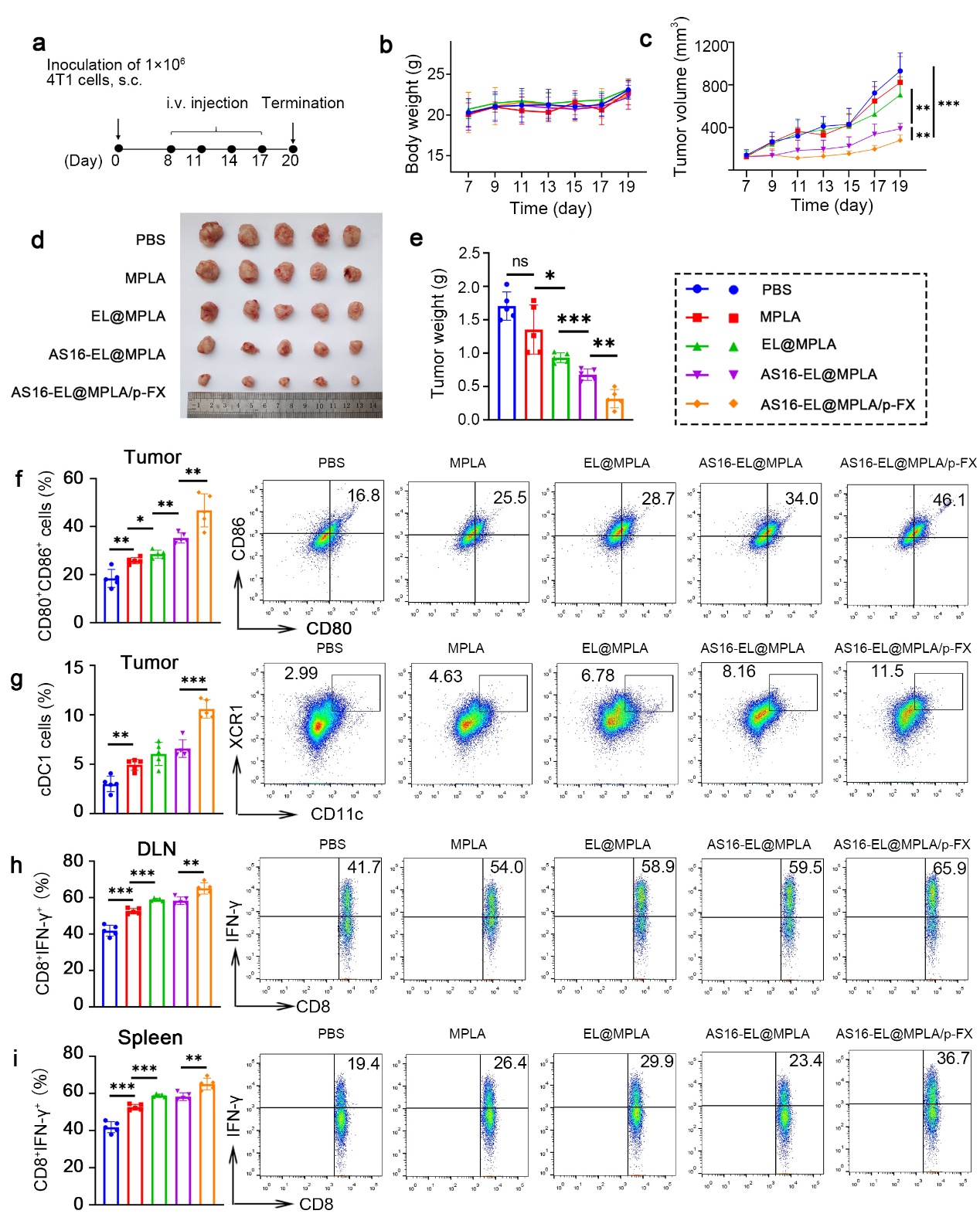


Figure S12. Antitumor efficacy of AS16-EL@MPLA/p-FX in 4T1-bearing mice. (a) Schematic diagram of AS16-EL@MPLA/p-FX used to treat the 4T1 tumor model. (b) Variations in the bodyweights of mice during the treatment period. (c) Changes in tumor volume in the different mouse groups. (d) Images of tumor tissues and (e) Tumor weights in the different mouse groups. (f) Flow-cytometric analysis of cells isolated from the tumors determined the percentage of maturation DCs with markers CD80, CD86. (g) Flow-cytometric analysis of cells isolated from the tumors determined the percentage of cDC1s in the tumors. (h,i) Flow-cytometric determined the percentage of IFN-γ expressing CD8^+^ T cells in draining lymph nodes and spleen. Data are means ± SD, *n* = 5; **p* < 0.05, ***p* < 0.01, ****p* < 0.001, ns: no significant difference.


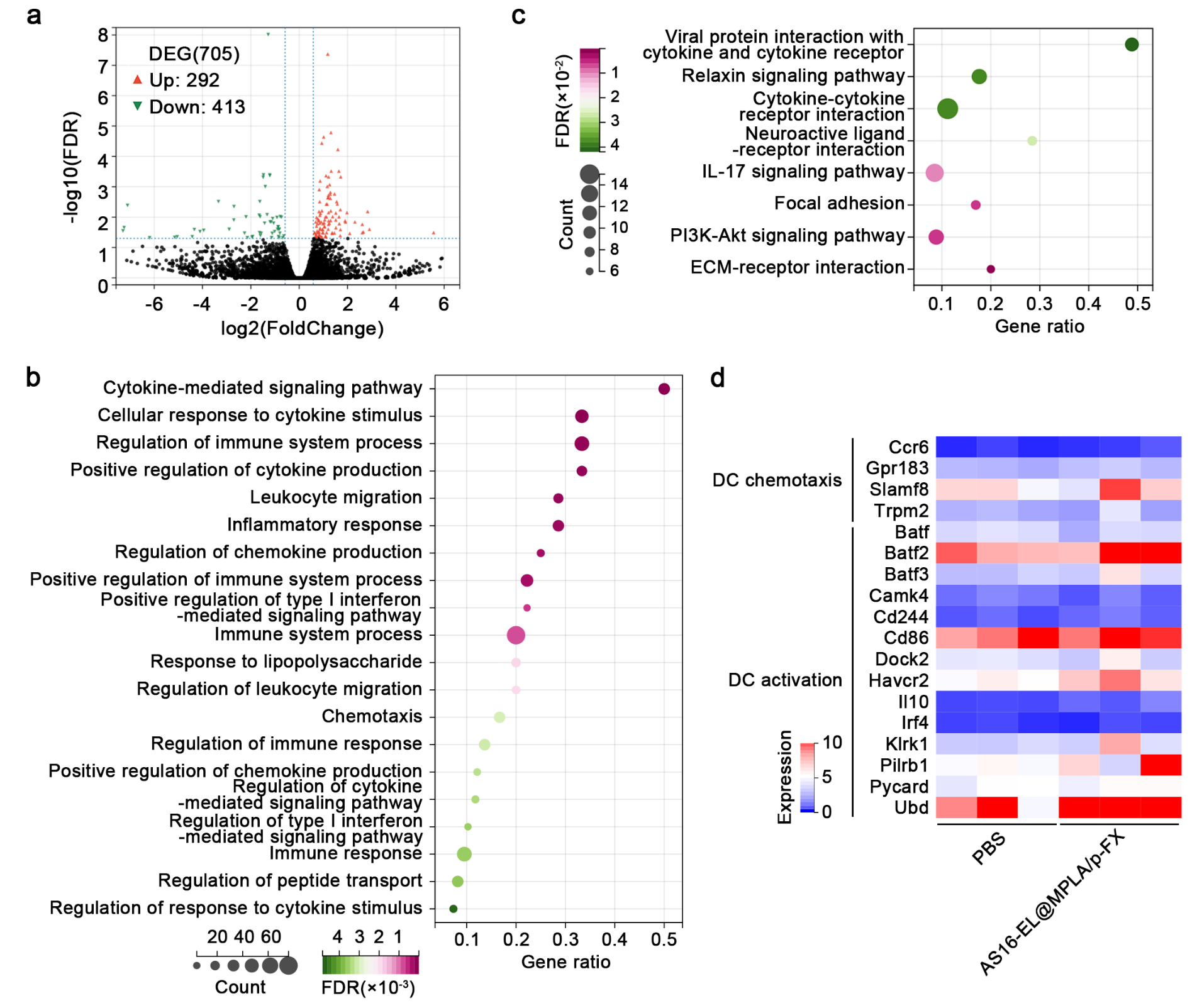


Figure S13. AS16-EL@MPLA/p-FX treatment reprograms the tumor immune landscape through cytokine-driven and chemokine-guided pathways. (a) Volcano plots of RNA-seq analysis. Red: upregulated genes; green: down-regulated genes. (b) GO enrichment network based on RNA-seq data analysis of upregulated genes. (c) Bubble chart indicating the enriched KEGG pathways of DEGs in AS16-EL@MPLA/p-FX *vs* PBS group. (d) Heatmap of representative genes related to DC chemotaxis and activation. *n* = 3


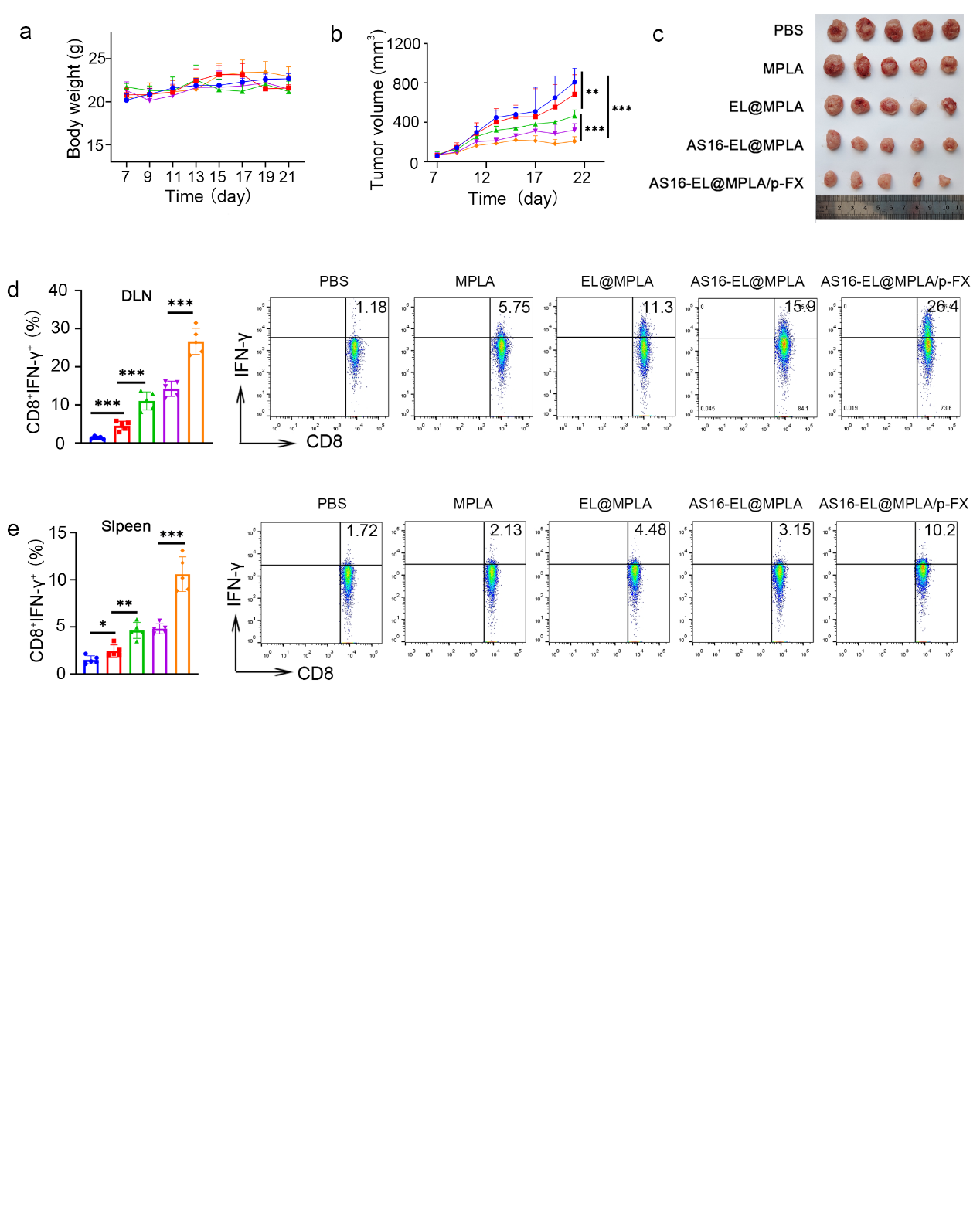


Figure S14. Antitumor efficacy of AS16-EL@MPLA/p-FX in 4T1-bearing mice. (a) Variations in the bodyweights of mice during the treatment period. (b) Changes in tumor volume in the different mouse groups. (c) Images of tumor tissues.


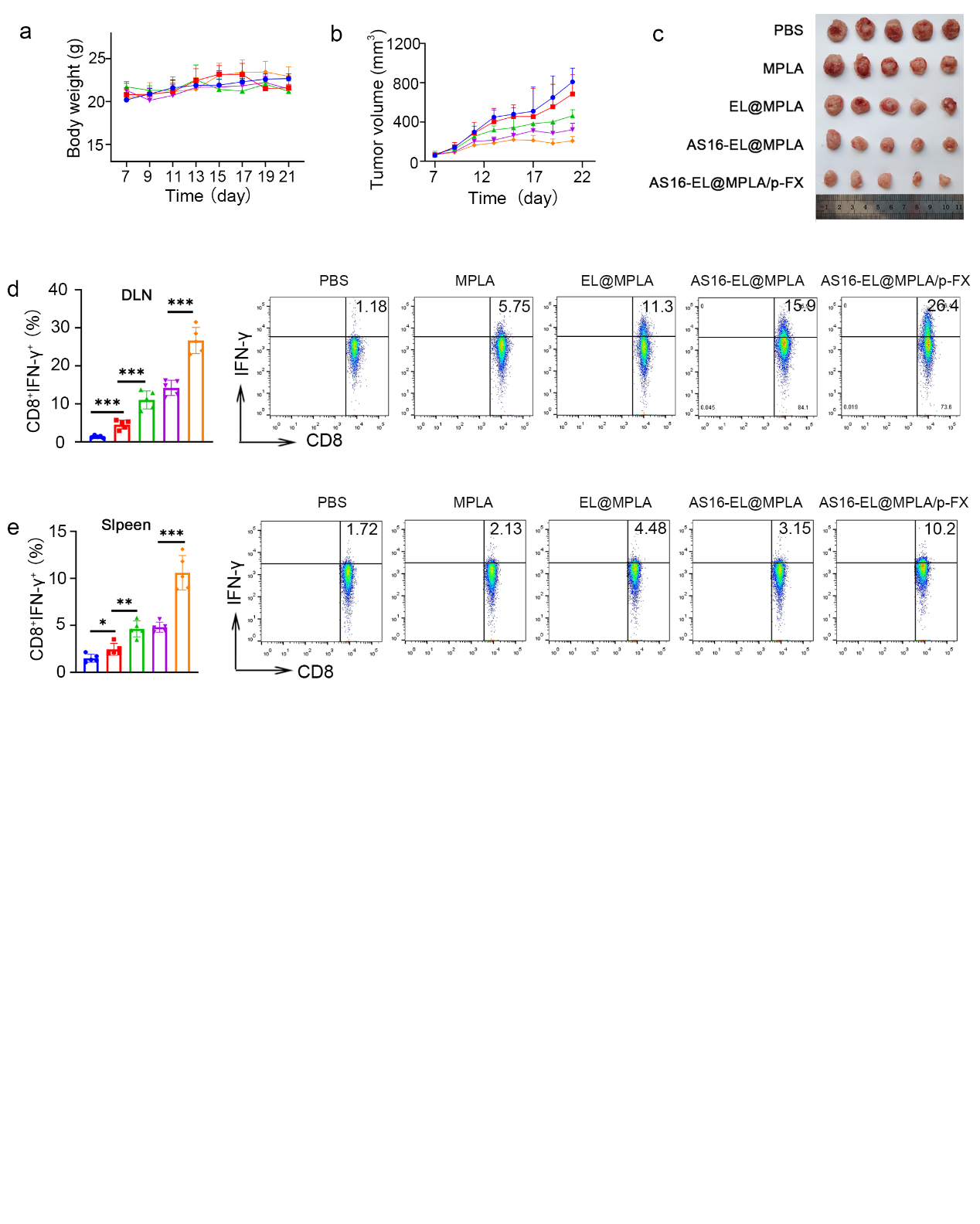


Figure S15. Flow-cytometric determined the percentage of IFN-γ expressing CD8^+^ T cells in draining lymph nodes. *n* = 5


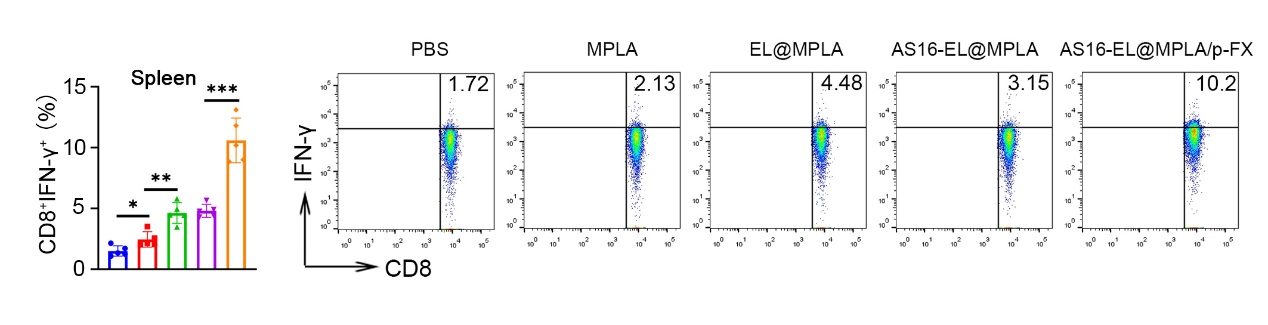


Figure S16. Flow-cytometric determined the percentage of IFN-γ expressing CD8^+^ T cells in spleen. *n* = 5


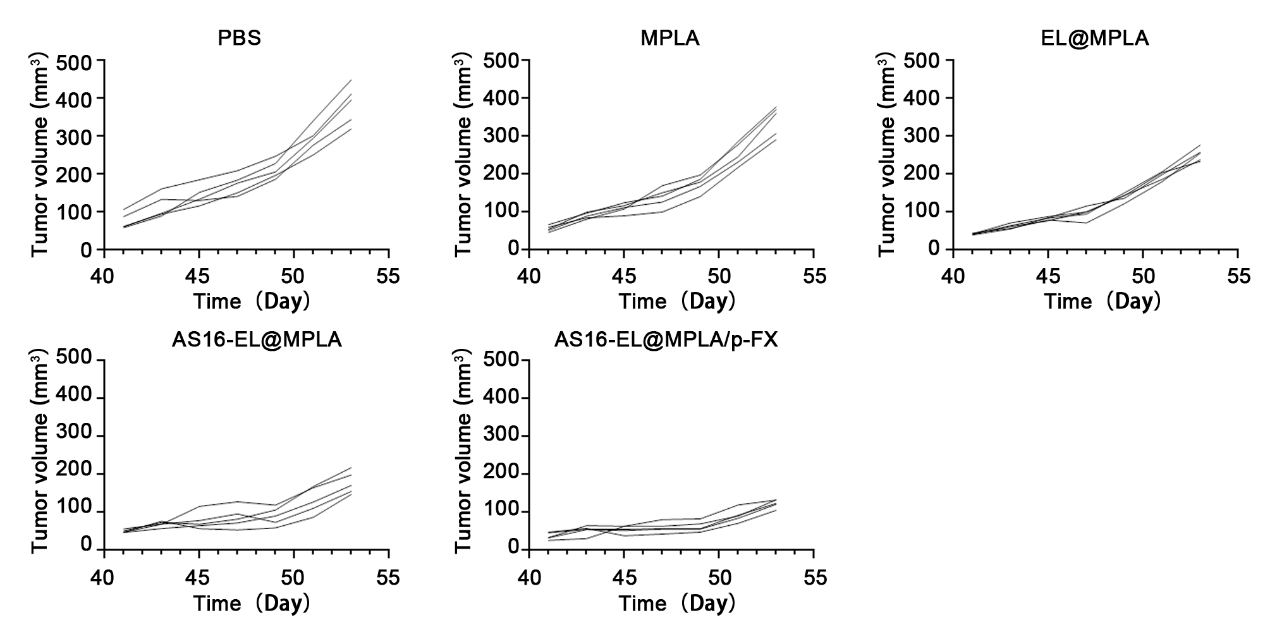


Figure S17. AS16-EL@MPLA/p-FX inhibited growth in rechallenged MC38 model. Each curve represents the growth of a single tumor in an individual mouse. Data are presented as the mean ± SD, *n* = 5.


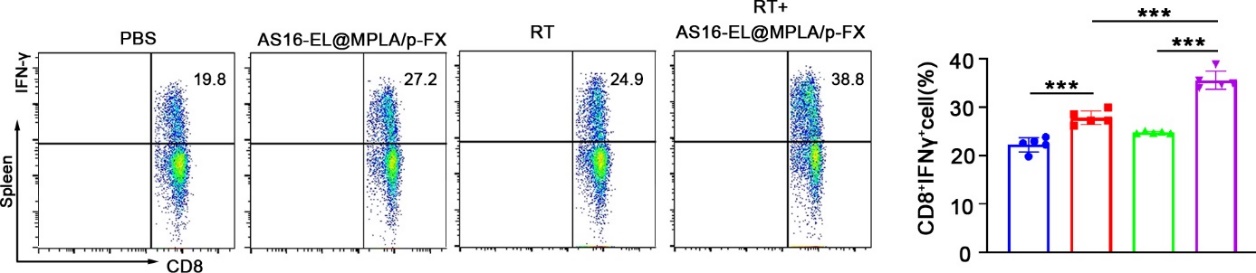


Figure S18. Representative flow plots depicting the percentages of CD8^+^ IFN-γ^+^ T cells in spleen. *n* = 5.


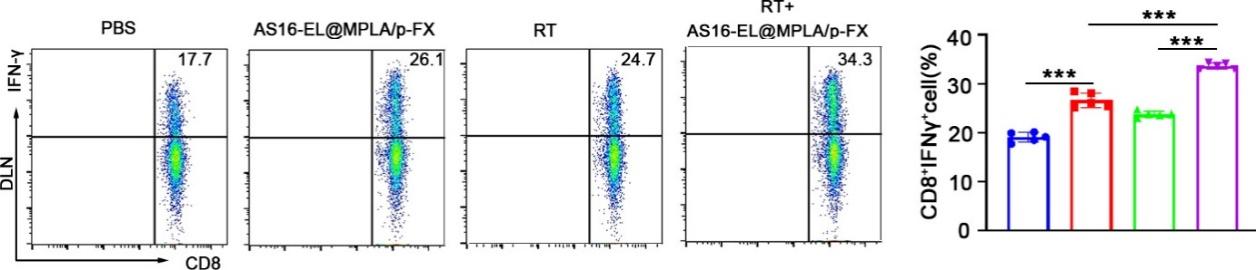


Figure S19. Representative flow plots depicting the percentages of CD8^+^ IFN-γ^+^ T cells in TDLN. *n* = 5.


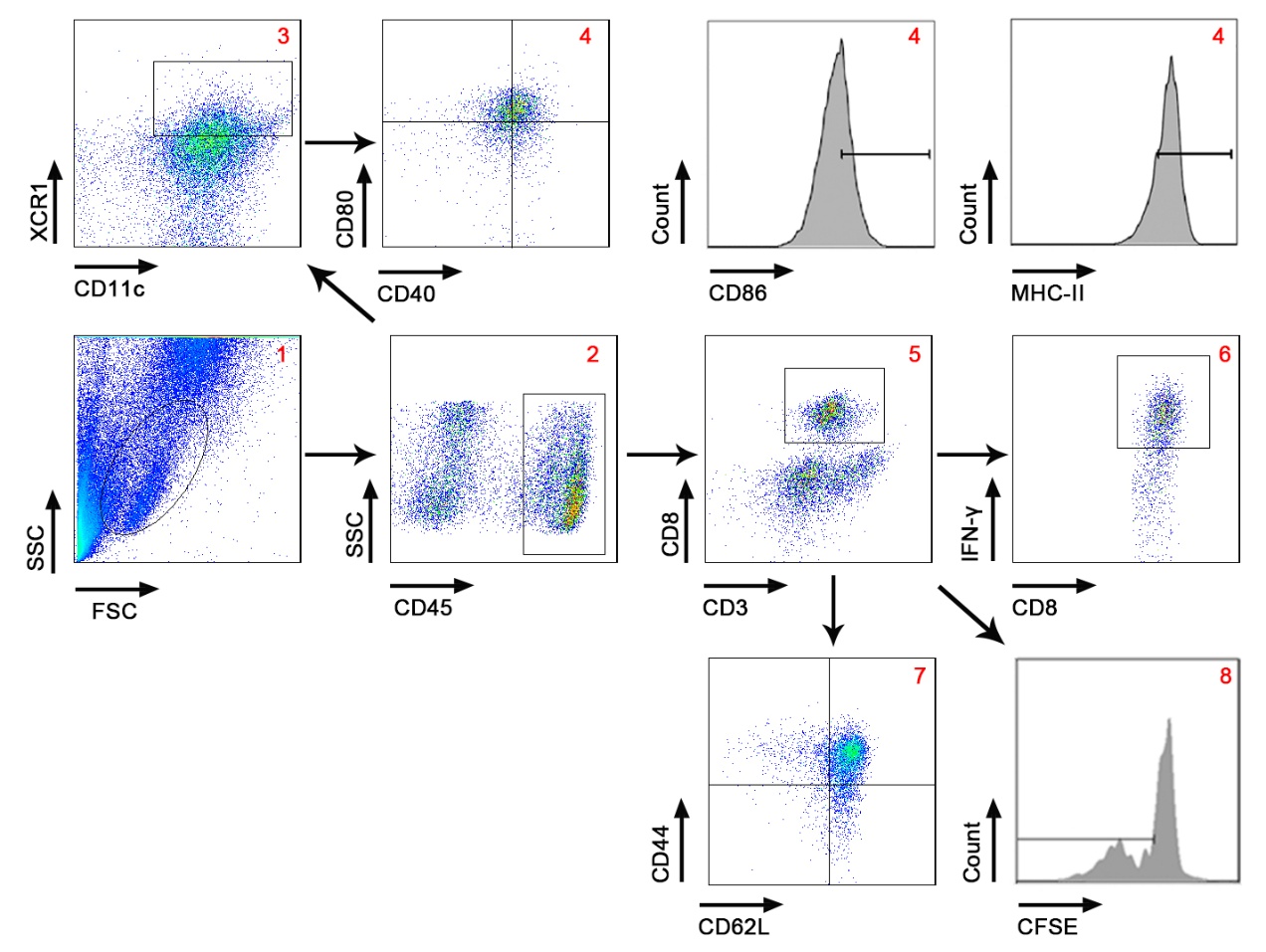


Figure S20. The complete gating strategy for flow cytometry analysis. Steps 1–4 were used to identify cDC1s (CD45⁺CD11c⁺XCR1⁺) and evaluate their activation (CD40, CD80, CD86, MHC-Ⅱ). Steps 1, 2, 5, 6, 8 were used to assess IFN-γ secretion and proliferation (CFSE^low^) of CD8⁺ T cells (CD45⁺CD3⁺CD8⁺). Steps 1, 2, 5, 7 were used to identify memory T cells (CD45⁺CD8⁺CD44⁺CD62L⁻).

Supplemental Table

Table S1. Drug loading (wt%) and encapsulation efficiency (EE%) of AS16-EL@MPLA/p-FX.

| Drug | wt% | EE% |
| --- | --- | --- |
| DNA | 63.93 ± 2.92 | 31.79 ± 1.79 |
| AS16 | 43.05 ± 4.05 | 4.30 ± 0.50 |
| MPLA | 37.93 ± 3.05 | 0.38 ± 0.03 |
